# Morphine‐mediated release of miR‐138 in astrocyte‐derived extracellular vesicles promotes microglial activation

**DOI:** 10.1002/jev2.12027

**Published:** 2020-11-19

**Authors:** Ke Liao, Fang Niu, Guoku Hu, Lu Yang, Blake Dallon, Delaney Villarreal, Shilpa Buch

**Affiliations:** ^1^ Department of Pharmacology and Experimental Neuroscience University of Nebraska Medical Center Omaha Nebraska USA

**Keywords:** drug abuse, extracellular vesicles, microglia, miRNA, neuroinflammation, opioids

## Abstract

Opioids, such as morphine, are the mainstay for the management of postsurgical pain. Over the last decade there has been a dramatic increase in deaths related to opioid overdose. While opioid abuse has been shown to result in increased neuroinflammation, mechanism(s) underlying this process, remain less understood. In recent years, microRNAs have emerged as key mediators of gene expression regulating both paracrine signaling and cellular crosstalk. MiRNAs constitute the extracellular vesicle (EV) cargo and can shuttle from the donor to the recipient cells. Exposure of human primary astrocytes to morphine resulted in induction and release of miR‐138 in the EVs isolated from conditioned media of cultured astrocytes. Released EVs were, in turn, taken up by the microglia, leading to activation of these latter cells. Interestingly, activation of microglia involved binding of the GUUGUGU motif of miR138 to the endosomal toll like receptor (TLR)7, leading, in turn, to cellular activation. These findings were further corroborated in vivo in wildtype mice wherein morphine administration resulted in increased microglial activation in the thalamus. In TLR7^−/−^ mice on the other hand, morphine failed to induce microglial activation. These findings have ramifications for the development of EV‐loaded anti‐miRNAs as therapeutics for alleviating neuroinflammation in opioids abusers.

## 1 INTRODUCTION

Opioids, such as morphine, are widely used prescription analgesics for acute postoperative pain and for moderate to severe pain, in both the emergency as well as primary care settings (Casamayor, Didonato, Hennebert, Brazzi, & Prosen, 2018; Haroutounian, 2018). In 2017, there were a total of 191 million opioid prescriptions dispensed, with ∼58.7 prescriptions per 100 people (CDC, 2018). It has been shown that even patients that undergo a short hospital stay for surgery are at an increased risk of long‐term opioid use resulting from dependence on opioid prescriptions (Alam, 2012). In the years 2007 to 2017, there was ∼2.6 fold increase in drug overdose deaths involving opioids ranging from 18,515 to 47,600 (NIH, 2019). Extensive opioid use was found to correlate with severity of central nervous system (CNS) complications, including memory impairment and depression (Sullivan, 2018; Terrett et al., 2014). Additionally, opioid such as morphine has also been shown to accelerate the rate of HIV‐1 infection as well as HIV‐associated neuroinflammation (Dave, 2012; El‐Hage et al., 2015; Li et al., 2003, Reddy, Pilakka‐Kanthikeel, Saxena, Saiyed, & Nair, 2012). The mechanism(s) underlying morphine‐mediated potentiation of CNS disease progression and pathogenesis, however, remain largely unknown.

Extracellular vesicles (EVs) play an important role in cell‐to‐cell communication both in cellular homeostasis as well as in disease pathogenesis (Caruso Bavisotto et al., 2019; Devhare & Ray, 2018; Men et al., 2019; Ñahui Palomino et al., 2019; Paolicelli, Bergamini, & Rajendran, 2019). It has been suggested that EV cargo comprising of proteins, lipids and RNAs contributes to the cellular crosstalk via receptor–ligand interactions (Russell et al., 2019; Sun et al., 2018; Tkach & Théry, 2016). In recent times, there has been an increased interest in exploring the source and cargo of EVs released during various pathologies such as cancer (Eylem et al., 2020; Joyce, Kerin, & Dwyer, 2016; Lewis et al., 2018; Liu et al., 2018), neurodegenerative diseases (Gámez‐Valero, Beyer, & Borràs, 2019; Shi et al., 2014), and viral infections (Liu et al., 2019; Rodrigues, Fan, Lyon, Wan, & Hu, 2018; Shen et al., 2017). In the CNS, EVs are found to be released by most cell types and have been implicated to play a key role in various neurodegenerative diseases, such as Alzheimer's Disease (Sardar Sinha et al., 2018), Parkinson's disease (Shi et al., 2014), Amyotrophic Lateral Sclerosis (Roy, Saucier, O'connell, & Morin, 2019) and HIV‐associated neurocognitive diseases (HAND) (Sampey et al., 2016). MiRNAs are small, evolutionarily conserved noncoding RNAs, that have been recognized as primary regulators in EV‐mediated cellular crosstalk in the CNS (Gupta & Pulliam, 2014; Men et al., 2019; Selmaj, Mycko, Raine, & Selmaj, 2017; Xia et al., 2019). For example, in our previous study, we reported that miR‐9 released from HIV protein transactivation of transcription (Tat)‐stimulated astrocytes could be taken up by the microglia, leading to enhanced microglial migration and, this involved targeted regulation of PTEN expression in the microglia. This study provided the evidence of glial crosstalk involving astrocyte‐derived EV (ADEV)‐miR9‐mediated migration of microglia in HAND (Yang et al., 2018). In addition to viral proteins, drugs of abuse such as opioids by themselves can also dysregulate the expression of miRNAs (Dutta & Roy, 2012; Guo et al., 2016; Hu et al., 2019; Hu et al., 2017; Periyasamy et al., 2018; Yang et al., 2018). An interesting study by Dave and Khalili showed that morphine‐mediated inflammation and oxidative stress induced in human monocyte‐derived macrophages contributed to the expansion of HIV‐1 viral reservoir in the CNS and HIV‐associated dementia (Dave, 2012). The authors provided evidence that this process was regulated by differentially expressed miRNAs, including miR‐15b & miR‐181b, both regulating several targets of the proinflammatory pathways. Similarly, findings from our lab have also reported that exosome‐mediated shuttling of miR‐29b regulates HIV Tat and morphine‐mediated neuronal dysfunction (Hu et al., 2012). Besides their ability to directly regulate gene transcription by binding to the 3’UTR region of the gene, some miRNAs can also directly bind to the toll‐like receptors (TLRs) via specific motifs (AU‐ and GU‐rich 4‐mers) present in their sequences (Gao, Shu, & Cui, 2018). As an example, let‐7 has been shown to induce neurodegeneration by binding to TLR7 receptor in both neurons and microglia (Lehmann et al., 2012). In keeping with this, studies by Yelamanchili *et. al*. have also demonstrated that HIV‐1 infection of blood‐derived macrophages resulted in significant upregulation of miR‐21 in EVs which, in turn, directly activated the TLR7‐dependent necroptosis pathway in neurons (Yelamanchili et al., 2015). Along these lines, our previous study has also demonstrated that EVs derived from morphine‐stimulated astrocytes could be taken up by microglia and activate the Toll‐like receptor 7 (TLR‐7), subsequently resulting in upregulated expression of long non‐coding (lnc)RNA‐Cox2, culminating ultimately into impaired microglial phagocytosis (Hu et al., 2018).

In the current study, we demonstrate the role of yet another miRNA, miR‐138, that is upregulated in the EVs released from morphine stimulated astrocytes which, in turn, was taken up by microglial cells, leading to the activation of microglia. This mechanism of uptake involved activation of the TLR7‐NF‐kB axis. Additionally, we also demonstrated that intranasal delivery of miR‐138 inhibitor alleviated microglial activation induced by morphine‐stimulated astrocyte EVs. Taken together these findings have ramifications for future development of EV‐loaded RNA‐based therapeutics for the treatment of neuroinflammatory diseases including opioids abuse disorders.

## 2 MATERIALS AND METHODS

### 2.1 Animals

C57BL/6N wild type (WT) mice (male, 6–8 weeks) were purchased from Charles River Laboratories, Inc. (Wilmington, MA). TLR7 knockout (KO) mice were purchased from Jackson Laboratories (Bar Harbor, ME, USA) and bred in the UNMC animal facility. Pregnant WT mice were purchased from Charles River Laboratories. All the animals were housed under conditions of constant temperature and humidity on a 12‐h light, 12‐h dark cycle, with lights on at 07:00 am. Food and water were available *ad libitum*. All animal procedures were performed according to the protocols approved by the Institutional Animal Care and Use Committee at the University of Nebraska Medical Center. Two‐month‐old male C57BL/6N mice were equally divided in two groups: saline and morphine. WT or TLR7 KO mice were randomly separated to two groups (*n* = 4), saline and morphine. The morphine group were injected intraperitoneally (i.p.) with morphine, three times a day every 8 h, at an initial dose of 10 mg/kg with an increment of 5 mg/kg every day for 6 d. Saline groups received a comparable volume of saline (as morphine group) daily. All mice were sacrificed 1 h after the last morphine/saline injection on the last day as reported previously (Cai et al., 2016).

### 2.2 Intranasal delivery of EVs in mice

For intranasal administration of EVs, we anesthetized C57BL/6N mice and placed them in a supine position in an anesthesia chamber. EVs (20 μg/100 μL) in saline were administered intranasally as drops with a small pipette every 2 min into alternating nostrils of the nasal cavity for a total of 10 min.

### 2.3 Cell cultures

The human astrocytic cell line A172 (no. CRL‐1620; American Type Culture Collection [ATCC]) was cultured as described previously (Hu et al., 2018) and maintained in DMEM with high glucose containing 10% heat‐inactivated fetal bovine serum (FBS), 2 mM glutamine, penicillin (100 U/ml), streptomycin (100 μg/ml). In this study, A172 cells were used within 30 passages. Human primary astrocytes were obtained from ScienCell Research Laboratories (Carlsbad, CA, USA) and were cultured in astrocyte medium (ScienCell). Human primary astrocytes were used within 10 passages in this study.

Mouse primary astrocytes were isolated from whole brains of post‐natal (1‐ to 3‐day‐old) C57BL/6N mice described as previously (Hu et al., 2017) and stained for astrocyte marker GFAP. Cells (> 97% GFAP positive) were plated onto poly‐D‐lysine pre‐coated cell culture flasks and maintained in DMEM (10% FBS, 100 U/ml penicillin, and 100 μg/ml streptomycin).

BV‐2 microglial cell line was generously provided by Dr. Sanjay Maggirwar (The George Washington University, School of Medicine and Health Sciences, Rochester, Washington, D.C., USA.) Cells were grown and routinely maintained in DMEM supplemented with 10% FBS, 100 U/ml penicillin, and 100 μg/ml streptomycin. BV‐2 cells were used under passage 20.

Mouse primary microglia cells were obtained from 1‐ to 3‐day‐old C57BL/6N or TLR7 KO newborn pups as described previously (Liao et al., 2016). After digestion and dissociation of the dissected brain cortices in Hank's buffered salt solution supplemented with trypsin (0.25%), mixed glial cultures were prepared by resuspending the cell suspension in DMEM supplemented with 10% heat‐inactivated FBS, OPI supplement, 100 U/ml penicillin, and 100 μg/ml streptomycin. Cells were plated at 20 × 10^6^ cells/flask density onto 75‐cm^2^ cell culture flasks with culture medium replaced every 5 days. Following the first medium change, macrophage colony‐stimulating factor (M‐CSF; 0.25 ng/ml; PeproTech, Rocky Hill, NJ, USA) was added to the flasks to promote microglial proliferation. The cell medium, containing released microglia cells, was collected from each flask and centrifuged at 800 *g* for 5 min to collect cells and plated onto cell culture plates.

HEK‐Blue TLR7 and HEK‐Blue Null cells were obtained from InvivoGen (San Diego, CA). Cells were cultured in DMEM supplemented with 10% heat‐inactivated FBS, 0.2% sodium bicarbonate, 100 IU/ml penicillin, 100 μg/ml streptomycin, 2 mM L‐glutamine, 30 μg/ml blasticidin, 100 μg/ml zeocin, and 100 μg/ml normocin at 37 °C in humidified air containing 5% CO_2_ as per the manufacturer's instructions.

### 2.4 Reagents

Morphine was purchased from R&D Systems (Minneapolis, MN, USA). Chemical inhibitors including the opioid receptor antagonist naltrexone, endosomal TLR inhibiter Chloroquin, IKK‐2 inhibitor SC514 were purchased from Sigma‐Aldrich (St. Louis, MO, USA). DOTAP Liposomal Transfection Reagent was also purchased from Sigma‐Aldrich. The mouse IL‐6 / TNFα DuoSet kits were obtained from R&D Systems (Minneapolis, MN, USA). Exo‐Fect^TM^ Exosome Transfection Kit was purchased from SBI System Bioscience (Palo Alto, Canada).

### 2.5 EV Isolation

EVs were prepared from the supernatant fluids (FBS depleted) of primary astrocytes and A172 cells by differential centrifugations as previously described (Hu et al., 2018). Briefly, conditioned media was harvested, centrifuged at 1000 × g for 10 min to eliminate cells, and again spun at 10,000 × g for 30 min, followed by filtration through a 0.22‐μm filter to remove cell debris. EVs were pelleted by ultracentrifugation (Beckman 32Ti rotor; Beckman Coulter, Brea, CA, USA) at 100,000 × g for 70 min. EVs were assessed for their protein content using a BCA Protein Assay Kit (Pierce, Rockford, IL, USA). Alix, TSG101 and CD63 were detected by western blotting as exosome markers. EVs were further quantified by Nanoparticle Tracking Analysis (NTA) using ZetaView.

### 2.6 Oligos and plasmid transfection

The RNA oligos (miR‐138 sequence: AGCUGGUGUUGUGAAUCAGGCCG; mutant‐miR‐138 sequence: AGCUGGUGAATUGAAUCAGGCCG) and cy5 labelled oligos were purchased from Integrated Technologies (Coralville, Iowa). EVs were loaded with oligos using Exo‐Fect Exosome Transfection Reagent according to the manufacturer's instructions. Anti‐miR138 were obtained from Integrated Technologies. pEF6. mCherry‐TSG101 was a gift from Quan Lu (Addgene plasmid 38318). Sequences of mouse Dicer1 siRNA oligonucleotides used in this study were: mouse Dicer1‐siS1, 5’‐GrUrGrUrCrArUrCrUrUrGrCrGrArUrUrCrUrArUrUr‐3’; mouse Dicer1‐si AS1, 5’‐UrArGrArArUrCrGrCrArArGrArUrGrArCrArCrUrUr‐3’; mouse Dicer1‐siS1, 5’‐CrCrArArCrUrArCrCrUrCrArUrArUrCrCrCrArUrUr‐3’; mouse Dicer1‐si AS2, 5’‐UrGrGrGrArUrArUrGrArGrGrUrArGrUrUrGrGrUrUr‐3’.

### 2.7 Western blotting

Brain tissues, treated cells or EVs were lysed using the Mammalian Cell Lysis kit (Sigma‐Aldrich), as described previously (Liao et al., 2016). Equal amounts of the proteins were electrophoresed in an SDS‐polyacrylamide gel under reducing conditions followed by transfering to PVDF membranes. Blots were blocked with 5% BSA in TBS‐Tween 20. The western blots were then probed with antibodies specific for Iba‐1 (1:1000; 019–19741; Wako), Tsg101 (1:1,000; ab125011; Abcam, Cambridge, MA, USA), Alix (1:1,000, ab117600; Abcam), CD63 (1:1,000; ab216130; Abcam), Flotillin (1:200, Cell Signaling Technology), Calnexin (1:1500; C7617; Sigma‐Aldrich), NF‐κB p65 (1:2,000; ab16502; Abcam), Histone H3 (1:1,000; 9715S; Cell Signaling Technology) and β‐actin (1:5,000; A5316; Sigma‐Aldrich). Secondary antibodies were alkaline phosphatase conjugated to goat anti‐mouse/rabbit IgG (1:10,000; Jackson ImmunoResearch Labs). Signals were detected by SuperSignal West Dura Extended Duration or Pico PLUS Chemiluminescent Substrate (Thermo Fisher Scientific, Waltham, MA). All experiments had at least four biological replicates, and representative blots are presented in the figures.

### 2.8 Real‐time PCR

For quantitative analysis of mRNA expression, comparative real‐time PCR was performed with the use of Taqman Universal PCR Master Mix (Applied Biosystems). Specific primers and probes for IL‐6, TNFα, GAPDH, mature miR‐138 and snRNA RNU6B (U6) were obtained from Applied Biosystems. All reactions were run in triplicate. The amount of miRNA was obtained by normalizing to snRNA RNU6B and relative to control as previously reported (Hu et al., 2017).

### 2.9 In situ hybridization and immunostaining

Human primary astrocytes were fixed and prehybridized in hybridization buffer (50% formamide, 10 mM Tris‐HCl, pH 8.0, 200 μg ml^−1^ yeast tRNA, 1 × Denhardt's solution, 600 mM NaCl, 0.25% SDS, 1 mM EDTA, 10% Dextran sulphate) at a concentration of 9 pM for the commercially available digoxigenin‐labelled miR‐138 probe (Exiqon, Woburn, MA). LNA‐modified miR‐138, labelled at both the 5′ and 3′ ends with digoxigenin (Exiqon), was diluted to a final concentration of 2 pM in hybridization buffer, heated to 65 °C for 5 min and separately hybridized to the brain sections at 37 °C overnight. The slides were then washed twice in hybridization buffer (without probe) at 37 °C, followed by washing three times in 2 × SSC and twice in 0.2 × SSC at 42 °C, followed by blocking with 1% bovine serum albumin, 3% normal goat serum in 1 × PBS for 1 h at room temperature and incubation with anti‐digoxigenin conjugated with horseradish peroxidase (1:100, Roche Diagnostics, Mannheim, Germany) and anti‐GFAP (1:400, G3893, Sigma‐Aldrich) antibodies overnight at 4 °C. The slides were washed twice with PBS and incubated with Alexa Fluor 488 goat anti‐rabbit IgG (1:400, Invitrogen, Carlsbad, CA) antibody for 1 h at room temperature. This was followed by two PBS washes and signal amplification (for the in situ, now labelled with horseradish peroxidase) using TSA Cy5 kit (PerkinElmer, Waltham, MA) according to the manufacturer's protocol. The slides were mounted in Prolong gold anti‐fade reagent with DAPI (Invitrogen, San Diego, CA). The specificity of the miR‐138 signal in FISH experiments was confirmed when compared with a scrambled control. Unlike the miR‐138, the scramble probe showed no signal in astrocytes.

### 2.10 RNA Immunoprecipitation

BV‐2 cells were transfected with the indicated miRNAs using Dotap and incubated at 37°C for 20 min. Cells were then extensively washed with 1.5 ml of ice‐cold PBS, collected and lysis performed through a 5 min incubation with 150 μl of Polysome lysis buffer on ice. Lysates were finally frozen in dry ice for 1 h, and then harvested at 14,000 × *g* for 15 min. 100 μl of each lysate was added to 50 μl of A/G protein (Santa Cruz, Dallas, TX), which was previously preincubated with 25 μl of NT2 buffer 5% BSA for 1 h and then with 100 μl of anti‐TLR7 antibody in rotation at 4°C overnight. Lysates were further incubated with beads (extensively washed with NT2 buffer, according to the manufacturer's protocol) (Myltenyi, Waltham, MA) in a final volume of 850 μl of NT2 buffer supplemented with 1 μl of 1M DTT and 34 μl of 0.5 M EDTA. Immunoprecipitation was performed for 5 h at 4°C in rotation. Beads were washed 4x with NT2 buffer and were incubated with 100 μl of NT2 buffer and Proteinase K (Qiagen, Germantown, MD) at 55 °C for 30 min, then RNA was extracted with Trizol and processed for real‐time analysis.

### 2.11 Biotinylated miRNA Pull‐Down

BV‐2 cells were transfected with 5’‐biotinylated mature miR‐138 and mut‐miR‐138 (20 μM final concentration) by Lipofectamine RNAiMax for 24 h. For a 10 cm dish, 50 μL of pre‐washed and blocked streptavidin agarose beads (Thermo Scientific) were incubated with the cell lysates at room temperature for 2 h, according to the manufacturer's instructions. Beads were washed for three times and followed by adding 50 μL 2X loading buffer. The samples were heated for 5 min at 95°C and subjected to western blotting for detection of TLR7 using anti‐TLR7 antibody.

### 2.12 Quanti‐Blue® SEAP reporter assay

HEK‐Blue mTLR7 cells are stably transfected with mTLR7, while HEK‐Blue Null1‐v is the parental cell line of HEK‐Blue mTLR7 (does not express mTLR7). Both cells were stably transfected with the secreted alkaline phosphatase (SEAP) reporter gene under the transcriptional control of an NF‐kB response element. The level of SEAP protein released into the culture media was used to quantify the extent of TLR7 stimulation, which also represents the levels of NF‐κB activation (Miller et al., 2020). HEK‐Blue‐Null1‐v and HEK‐Blue mTLR7 cells (1 × 10^4^) were plated in 96‐well plates and grown to 70% confluence. Cells were then transfected with Dotap‐miR138, Dotap‐mut‐miR138, EV‐miR138, EV‐mut‐miR138 for 16 h. Cells were also treated with CL294, a TLR7 agonist, as a positive control. Aliquots of the culture medium (20 μl) were removed and added to new 96‐well plates containing 180 μl of pre‐warmed Quanti‐Blue™, a SEAP colorimetric detection medium, as per manufacturer's instructions. Color was allowed to develop for 1 h, and absorbance was read at 650 nm in a Bio‐Tek ® microplate reader (Burlington, VT).

### 2.13 IL‐6/TNFα protein analysis by ELISA

Culture supernatants were collected from mouse primary microglia exposed to various treatments and assessed for expression of IL‐6 / TNFα protein using the mouse IL‐6 DuoSet Kit or mouse TNF‐α DuoSet Kit (R&D Systems, Minneapolis, MN).

### 2.14 Immunostaining and image analysis

WT or TLR7 KO mice (*n* = 4) were euthanized 1 h post‐injection and brains were processed for embedding in paraffin. Formalin‐fixed, paraffin‐embedded (FFPE) blocks were sectioned at 4 μm and stained with antibody specific for Iba1 (1:250; Wako) overnight at 4°C. Next day, sections were washed with PBS for three times followed by incubation with biotinylated goat anti‐rabbit immunoglobulin G (1:200) in immunoblocking buffer at RT for 1 h and incubated with an avidin‐biotin‐peroxidase kit for 1 h. Horseradish peroxidase reaction product was visualized with enhanced DAB peroxidase substrate kit. The sections were scanned with the Ventana iScan HT slide scanner (Roche, Basel, Switzerland) at 40X magnification. The morphology of microglia in various regions including cortex, hippocampus, thalamus, striatum was quantified by Image J followed by the method described previously (Fernández‐Arjona, Grondona, Granados‐Durán, Fernández‐Llebrez, & López‐Ávalos, 2017; Young & Morrison, 2018). Five or six cells per animal were used for the quantification.

Mouse primary microglia or BV2 cells cultured on coverslips were fixed with 4% formaldehyde in PBS for 20 min at RT. The slides or coverslips were washed three times with PBS, permeabilized with 0.3% Triton X‐100 for 30 min, rewashed three times, and blocked in 10% goat serum in PBS for 2 h at RT. The following antibodies were used for immunostaining: Iba1 (1:250; Wako, Richmond, VA), EEA1 (1:100; #3288; Cell signaling, Danvers, MA), NF‐κB (1:100; ab16502; Abcam), TLR7 (1:100; ab45371; Abcam, Cambridge, MA, 1:1000; NBP2‐27332; Novus Biologicals, CO). The slides or coverslips were washed with PBS and incubated with Alexa Fluor 488–conjugated anti‐rabbit or anti‐mouse (Invitrogen, San Diego, CA) for 1 h at RT. After final washing with PBS, the slides or coverslips were mounted with mounting medium (Prolong Gold Antifade Reagent; Invitrogen). Fluorescent images were acquired at RT on a Zeiss Observer, using a Z1 inverted microscope with a 40 × /1.3 or 63 × /1.4 oil‐immersion objective. Images were processed with the AxioVs 40 Version 4.8.0.0 software (Zeiss, San Diego, CA). Photographs were acquired with an AxioCam MRm digital camera and were analyzed with ImageJ software.

### 2.15 Adult microglia isolation

Microglia were isolated from whole brain homogenates by Percoll gradient centrifugation as reported previously (Guo et al., 2016) with slight modifications. Briefly, the brains were homogenized in PBS (pH 7.4) by passing through a 70‐μm nylon cell strainer. Resulting homogenates were centrifuged at 600× *g* for 6 min. Supernatants were removed and cell pellets were resuspended in 70% isotonic Percoll (GE‐healthcare, Uppsala, Sweden) at room temperature. A discontinuous Percoll density gradient was layered as follows: 70, 50, 35 and 0% isotonic Percoll. The gradient was centrifuged for 20 min at 2000 × *g* and microglia were collected from the interphase between the 70 and 50% Percoll layers. Cells were washed and then resuspended in sterile PBS followed by flow cytometry analysis by gating the myeloid cells for the CD11b^+^/CD45^low^ population.

### 2.16 Statistical analysis

Statistical analysis was performed using a two‐tailed Student's t test for comparison of the two groups and one‐way ANOVA with a Bonferroni's post hoc test for multiple comparisons. For comparison between the two groups, an F test was used to determine the equality of variances between groups. For comparison among multiple groups, a Brown‐Forsythe test was used to determine the equality of variances among groups. Results were judged statistically significant if *P* < 0.05 by ANOVA for both Student's t test and one‐way ANOVA test. Data distribution was assumed to be normal, but this was not formally tested.

## 3 RESULTS

### 3.1 Morphine‐mediated changes in microglial morphology

**FIGURE 1:**
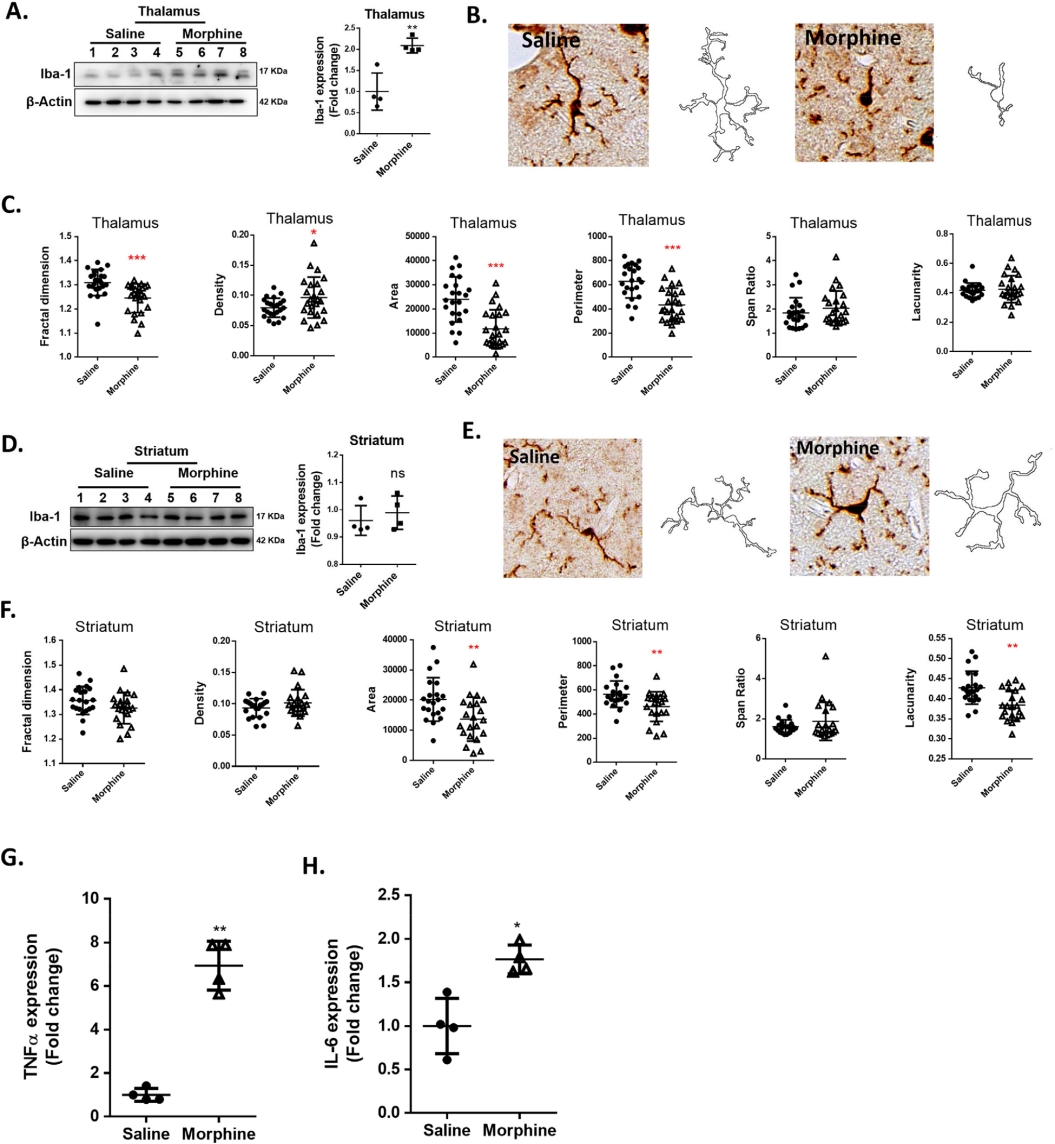
Morphine‐induced microglial activation. (A) Representative western blot and quantification of Iba1 in thalamus lysates from mice administrated saline or morphine (*n* = 4/group). (B) Representative images of Iba‐1+ cells and pairwise outline shapes were used for morphological parameter measures in the thalamus of mice administrated saline or morphine. (C) Quantification of morphological parameters such as fractal dimension, density, area, perimeter, span ratio and lacunarity in the thalamus of mice administrated saline or morphine (*n* = 5 or 6 cells/animal). (D) Representative western blot and quantification of Iba1 in the lysates of striatum of mice administrated saline or morphine (*n* = 4/group). (E) Representative images of Iba‐1+ cells and pairwise outline shapes were used for morphological parameter measures in the striatum of mice administrated saline or morphine. (F) Quantification of morphological parameters: fractal dimension, density, area, perimeter, span ratio and lacunarity in the striatum of mice administrated saline or morphine (*n* = 5 or 6 cells/animal). (G and H) Real‐time PCR analysis of TNF‐α mRNA (G) and IL‐6mRNA(H) expression in adult microglia cells isolated from the brains of mice administrated saline or morphine (*n* = 4/group). All data are presented as mean ± SD or SEM of three or four individual experiments. *,*P* < 0.05; **,*P* < 0.01; ***,*P* < 0.001 versus saline group using Student's t test.

Microglial morphology is closely related to its function and activation status (Fernández‐Arjona et al., 2017; Fernández‐Arjona, Grondona, Fernández‐Llebrez, & López‐Ávalos, 2019). Herein, we sought to determine morphine‐mediated activation of microglia by examining the expression of microglial protein Iba‐1 and the associated morphological changes. For this C57BL/6N mice were injected with morphine three times a day for 7 consecutive days as described previously, followed by assessment of microglial marker Iba1 expression by western blotting and examination of microglial morphology by immunostaining for Iba1, in various brain regions (cortex, hippocampus, thalamus and striatum). The expression of Iba‐1 was significantly upregulated in the thalamus (Figure 1a), but not in other brain regions (Figure 1d & SFigs.1A & D) of morphine‐administered mice compared with the saline group. The fractal dimension which determines the complexity of pattern was significantly decreased in the thalamus (Figure 1c), but not in the other regions (Figure 1f & SFigs.1C & F) of morphine‐administered mice compared with the saline group. Density of cells also referred to as the parameter solidity, was found to be significantly increased in the thalamus (Figure 1c), but not in other brain regions (Figure 1f & SFig.1C & F) of morphine‐administrated mice compared with the saline group. Area of cells was also found to be significantly decreased in the thalamus (Figure 1c), striatum (Figure 1f), and cortex (SFig.1C), but not in the hippocampus (SFig.1F) of morphine‐administrated mice compared with the saline group. Perimeter, which measures the single outline of cell shape was found to be significantly decreased in the thalamus (Figure 1c) and striatum (Figure 1f), but not in the cortex (SFig.1C) or hippocampus (SFig.1F) of morphine‐administrated mice compared with the saline group. Span ratio which measures the ratio of the major over the minor axes of the convex hull remained unchanged in all the regions. Additional morphological feature, Lacunarity, was found to be significantly changed only in the striatum (Figure 1f). To further assess the effects of morphine on microglial activation, ex vivo isolated adult microglial cells from morphine or saline administrated mice were isolated by Percoll gradient centrifugation and sorted by labeling with CD11b and CD45 using flow cytometry. CD11b^high^CD45^low^ microglia cells were collected and assessed for expression of proinflammatory cytokines such as TNFα/ IL‐6 by real‐time PCR. As shown in Figure 1g and h, expression of TNFα/ IL‐6 was found to be significantly increased in the adult microglia isolated from morphine‐administrated mice compared with the saline group.

### 3.2 Role of mu‐opioid receptor in upregulation of EV miR‐138 released from morphine‐stimulated astrocytes

The microglial morphology was initially tested to assess whether morphine could mediate activation of these cells. Once we observed that it could, we then rationalized, that based on the fact that in the CNS, ADEVs contribute to dysfunction of multiple cell types (Hu et al., 2012; Hu et al., 2018; Hu et al., 2020; Nogueras‐Ortiz et al., 2020; Venturini et al., 2019; Willis et al., 2020; Yang et al., 2018), that this effect on microglia, could also be involving the ADEVs. Hence after observing a phenomenon of microglial activation, we next sought to understand the role of ADEVs in this process. We first isolated EVs from the conditioned media (CM, FBS depleted) of mouse primary astrocytes, A172 – a human astrocytic cell line and human primary astrocytes, and characterized the EVs (Hu et al., 2018). As shown in SFig.2A, C and E, immunoblotting of the lysates obtained after various centrifugation steps (1000 × *g*, 10,000 × *g*, 100,000 × *g* pellets) identified the presence of signature exosomal markers Alix, CD63, and TSG101, in the EV lysates (100,000 × *g* pellet). Calnexin was used as a negative control to assess contamination (if any) of cell debris in ADEVs. As demonstrated the signal for calnexin was negative in all the EV lysates. Nanoparticle analysis using the ZetaView revealed a reasonable yield of EVs of the expected size (∼100 nm; SFigs. 2B, D and F). Atomic force microscopy (AFM) image clearly demonstrated that the diameter of the isolated EVs ranged from 40–100 nm (SFig. 2G).

The next step was to examine whether ADEVs could be taken up by microglia. For this mouse primary microglial (MPM) cells were cultured with ADEVs isolated from mouse primary astrocytes transfected with a plasmid encoding the exosomal marker TSG101 fused with mCherry. MPMs were then immunostained for the microglial marker Iba1 and as shown in SFig.3A, EVs‐TSG101‐mCherry were taken up by the microglial cells within 30 min. Next, we sought to assess whether EVs‐TSG101‐mCherry could reach the endosomes, the intracellular organelles which are the site for localization of TLR7. Following incubation with EVs‐TSG101‐mCherry (30 min) MPMs were immunostained with the endosomal marker EEA1 and, as shown in SFig. 3B, ADEVs were found to co‐localize with EEA1 in MPMs. These phenomena were matched with our previous findings (Hu et al., 2018).

**FIGURE 2:**
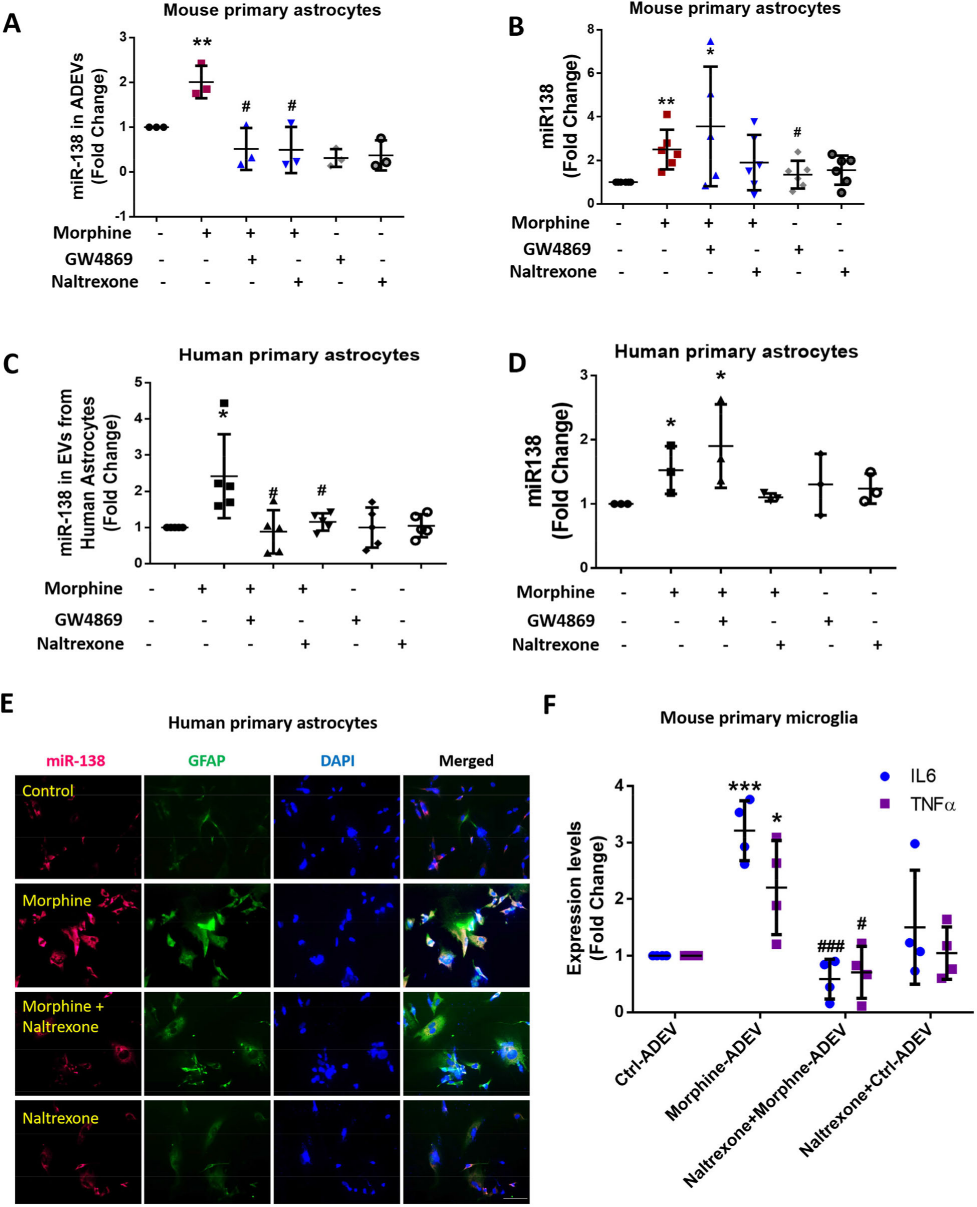
Morphine‐induced upregulation of miR138 in ADEVs involves mu receptor. (A) Real‐time PCR analysis of miR138 expression in EVs isolated from mouse primary astrocytes pretreated with the exosome release inhibitor GW4869 (10 μM) or mu receptor antagonist, Naltrexone (10 μM), followed by morphine exposure for additional 24 h. One‐way ANOVA followed by Bonferroni's post hoc test was used to determine the statistical significance among multiple groups (*n* = 3). (B) Real‐time PCR analysis of miR138 expression in mouse primary astrocytes pretreated with the exosome release inhibitor GW4869 (10 μM) or mu receptor antagonist Naltrexone (10 μM), followed by morphine exposure for additional 24 h. One‐way ANOVA followed by Bonferroni's post hoc test was used to determine the statistical significance among multiple groups (*n* = 6). (C)Real‐time PCR analysis of miR138 expression in EVs isolated from human primary astrocytes pretreated with exosome release inhibitor GW4869 (10 μM) or mu receptor antagonist Naltrexone (10 μM), followed by morphine exposure for additional 24 h. One‐way ANOVA followed by Bonferroni's post hoc test was used to determine the statistical significance among multiple groups (*n* = 5). (D) Real‐time PCR analysis of miR138 expression in human primary astrocytes pretreated with exosome release inhibitor GW4869 (10 μM) or mu receptor antagonist Naltrexone (10 μM), followed by morphine exposure for additional 24 h. One‐way ANOVA followed by Bonferroni's post hoc test was used to determine the statistical significance among multiple groups (*n* = 3). (E) Representative images of ISH assay using a probe specific for miR‐138 (red) combined with an immunostaining for astrocyte marker GFAP (green) in human primary astrocytes. Scale bar, 10 μm (*n* = 3). (F) Real‐time PCR analysis of IL‐6 / TNFα mRNA expression in mouse primary microglia exposed to Morphine‐ADEV isolated from astrocytes pretreated with mu receptor antagonist Naltrexone (10 μM), followed by morphine exposure for additional 24 h. One‐way ANOVA followed by Bonferroni's post hoc test was used to determine the statistical significance among multiple groups (*n* = 4). All data are presented as mean ± SD or SEM of at least three individual experiments. *,*P* < 0.05; **,*P* < 0.01; ***,*P* < 0.001 versus control group. #,*P* < 0.05; ###,*P* < 0.001 versus morphine group.

In our previous study, using RNA sequencing (RNA seq) we had identified 15 of the 4‐mer containing miRNAs (that function as TLR7/8 RNA agonists) to be significantly upregulated in morphine‐ADEVs compared with control‐ADEVs (Ctrl‐ADEVs). Out of the upregulated miRs, miR‐138 was chosen based on its significant upregulation and increased abundance in the EVs among all the other miRs (Hu et al., 2018). We next sought to examine the expression of miR‐138 in both ADEVs and intracellular in the astrocytes exposed to morphine. As shown in Figure 2a and b, there was increased expression of miR‐138 both in ADEVs as well as in mouse primary astrocytes (MPAs) in response to morphine exposure. Interestingly, pre‐treatment of astrocytes with naltrexone (opioid receptor antagonist) blocked morphine‐mediated upregulation of miR‐138 in both ADEVs and in MPAs (Figure 2a and b). Furthermore, as expected, pretreatment of MPAs with GW4869 (exosome release inhibitor) alleviated morphine‐mediated upregulation of miR‐138 in ADEVs, but not intracellularly in the cells. Similar phenomenon of miR138 upregulation in EVs and in the cells was also validated in human primary astrocytes (Figure 2c and d). These findings were further validated by in situ hybridization (ISH) using DIG labeled‐miR‐138 probe, wherein increased expression of miR‐138 RNA was observed in morphine‐stimulated human primary astrocytes compared with untreated cultures (Figure 2e). Pretreatment of human primary astrocytes with naltrexone mitigated morphine‐mediated upregulation of miR‐138 (Figure 2e). We next sought to examine whether morphine‐ADEVs played a role in microglial activation, and if so, whether this process involved the mu‐opioid receptor. Briefly, ADEVs were isolated from conditioned media of mouse primary astrocytes treated with morphine (10 μM) for 24 h. MPMs were then exposed to either morphine or control ADEVs at a concentration of 500 EVs/cell followed by isolation of cellular RNA which was then assessed for the expression of IL‐6 and TNFα by real‐time PCR. As shown in Figure 2f, exposure of MPMs to morphine‐ADEVs resulted in upregulated expression of both IL‐6 and TNFα. To examine the effect of mu‐opioid receptor on morphine‐ADEVs‐mediated upregulation of IL‐6 and TNFα expression, mouse primary astrocytes were pretreated with naltrexone (10 μM; 1 h), followed by exposure of cells to morphine (24 h). Subsequently, naltrexone‐Morphine‐ADEVs and naltrexone‐Control‐ADEVs were isolated from the conditioned media and exposed to MPMs. Naltrexone‐Morphine‐ADEVs failed to induce the expression of IL‐6 and TNFα in MPMs.

### 3.3 ADEV miR‐138 interacts with murine TLR7 in the endosomes

**FIGURE 3:**
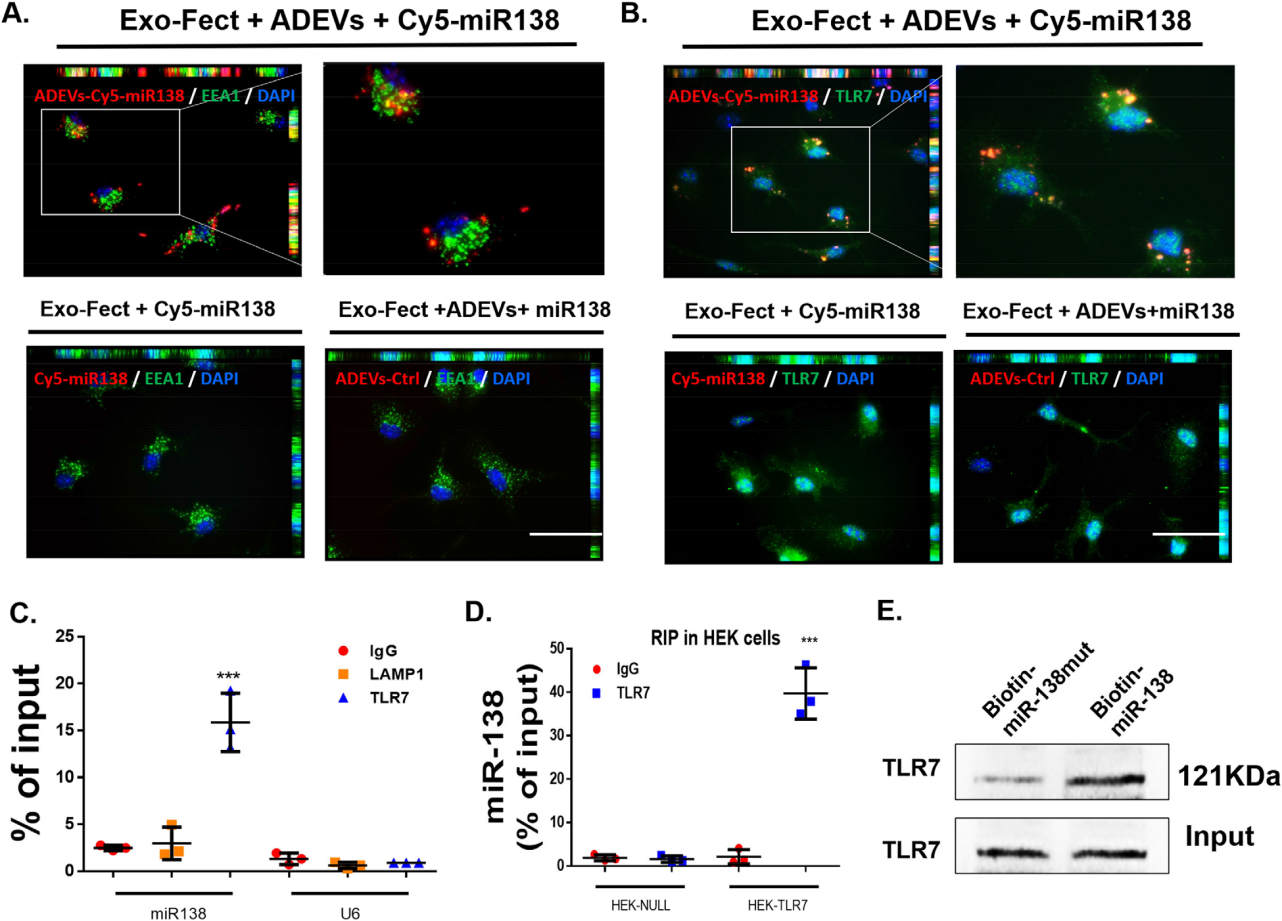
ADEV miR‐138 interacts with murine TLR7 in the endosomes. (A) Representative fluorescence images of mouse primary microglial cells incubated with Exo‐Fect+ADEVs+Cy5‐miR138 or Exo‐Fect+Cy5‐miR138 (without ADEVs) or Exo‐Fect+ADEVs+miR138 (unstained) for 30 min followed by immunostaining of (A) an early endosome marker (EEA1, Green) and (B) TLR7 (Green). Exo‐fect+ADEVs+Cy5‐miR138, Cy5‐miR138 were loaded into ADEVs using by Exo‐Fect transfection kit; Exo‐fect+Cy5‐miR138, Cy5‐miR138 were loaded using by Exo‐Fect transfection kit (without ADEVs); Exo‐fect+ADEVs+miR138, unstained miR138 were loaded into ADEVs using Exo‐Fect transfection kit; Bars, 50 μm (*n* = 3). (C) TLR7 was immunoprecipitated from BV2 cells by IgG / TLR7 / LAMP1 antibody, followed by assessment of miR138 / U6 expression by real‐time PCR. One‐way ANOVA followed by Bonferroni's post hoc test was used to determine the statistical significance among multiple groups (*n* = 3). (D) TLR7 was immunoprecipitated from HEK‐Null / HEK‐TLR7 cells by IgG/TLR7 antibody, followed by assessment of miR138 expression by real‐time PCR. One‐way ANOVA followed by Bonferroni's post hoc test was used to determine the statistical significance among multiple groups (*n* = 3). (E) The protein of TLR7 were pull down by miR‐138‐biotin / miR‐mut‐138‐biotin with Streptavidin agarose beads in BV2 cells. All data are presented as mean ± SD or SEM of three individual experiments. *,*P* < 0.05; **,*P* < 0.01; ***,*P* < 0.001 versus control group.

The first step here was to examine whether ADEVs‐miR138 could reach the endosomes, and if so, whether ADEVs‐miR138 could bind to TLR7. Briefly, we first loaded ADEVs with cy5 labeled miRNA‐138 using Exo‐Fect kit (Exo‐Fect+ADEVs+cy5‐miR‐138). To assess whether this method could successfully load cy5‐miR138 into the ADEVs (and was not present on the surface of ADEVs), mouse primary microglial cells were exposed to ADEV‐Cy5‐miR138+RNaseA (Cy5‐miR138 was first loaded into the ADEV using Exo‐Fect, followed by the incubation with RNaseA) or ADEV+RNaseA‐Cy5‐miR138 (Cy5‐miR138 was first incubated with RNaseA followed by loading into ADEVs using Exo‐Fect), and subsequently immunostaining MPMs for the endosomal marker EEA1. As shown in SFig. 3C, in the group where Cy5‐miR138 was first loaded into the ADEV followed by incubation with RNaseA, we found Cy5‐miR138 colocalized with the endosomal EEA1. However, in the group of Cy5‐miR138 was first incubated with RNaseA followed by loading into ADEVs, no Cy5‐miR138 signals were observed. Our results thus indicated that the Cy5‐miR138 was located inside the EVs after loading Cy5‐miR138 into ADEVs using the Exo‐fect transfection kit. Mouse primary microglial cells were then exposed to Exo‐Fect+ADEVs+Cy5‐miR138 for 30 min followed by immunostaining MPMs for the endosomal marker EEA1. As shown in Figure 3a, there was colocalization of ADEVs‐Cy5‐miR138 with the endosomal marker EEA1. In the absence of ADEVs, Cy5‐miR138 could not be delivered into MPMs using the Exo‐Fect transfection reagent. Thus, Cy5‐miR138 failed to colocalize with the endosomes. ADEVs loaded with unstained miR‐138 was used as a negative control. Having demonstrated that ADEVs containing miR138 could be taken up by MPMs and reach the endosomal compartment, the next step was to examine whether ADEVs‐Cy5‐miR138 colocalized with TLR7 on the endosomes. MPMs exposed to Exo‐Fect‐ADEVs‐Cy5‐miR‐138 were subsequently immunostained for TLR7 and as shown in Figure 3b, there was colocalization of ADEVs‐Cy5‐miR138 with TLR7. Exo‐Fect+Cy5‐miR138 (without ADEVs) or ADEVs loaded with unstained miR138(Exo‐Fect‐ADEVs‐miR138) served as negative controls.

The next logical step then was to examine the binding of miR‐138 to endosomal TLR7 by RNA immunoprecipitation (RIP) assay. Lysates of BV2 microglial cells that were first immunoprecipitated with the antibody specific for TLR7 or LAMP1 (lysosomal protein, negative control), followed by assessing for expression of miR138 in the immunoprecipitants by real‐time PCR. As shown in Figure 3c, miR‐138 was highly enriched in the TLR7 co‐immunoprecipitated complex compared with the IgG control and LAMP1 co‐immunoprecipitated complex. Further validation of these findings was done in HEK‐TLR7 cells and similar to BV2 cells, there was enrichment of miR‐138 in the TLR7 co‐immunoprecipitated complex in HEK‐TLR7 cells compared with the HEK‐Null cells (Figure 3d). Validation was also done by pull down assays, where BV2 cells were transfected with biotin‐labelled miR138 or biotin labeled mutant miR‐138 followed by pull down using Streptavidin agarose beads. As shown in Figure 3e, there was enrichment of TLR7 in miR‐138‐biotin complex compared with lysates from miR‐mut‐138‐biotin transfected cells. These findings alluded to a possible interaction between miR‐138 and TLR7.

### 3.4 ADEV miR‐138 mediated nuclear translocation of NF‐κB p65 involves TLR7

Having determined the binding of miR‐138 to TLR7, the next step was to assess the functionality of this binding. Herein, Dotap (a liposomal cationic lipid used to generate liposomes; an EV mimic (Fabbri et al., 2012)) was used to encapsulate and directly deliver miR‐138 to BV2 cells. To examine the specificity of miR functionality in the recipient BV2 cells, it was important to first examine the delivery of miRNA into these cells. BV2 cells were transfected with Dotap containing Cy5‐miR138 for 30 min followed by immunostaining the cells with Iba1, EEA1 and TLR7. Similar to the exposure of MPMs to ADEVs‐Cy5‐miR‐138 (Figure 3), Dotap‐Cy5‐miR138 was also found to accumulate in the cytoplasm of BV2 cells (SFig. 4A) and co‐localized with microglial endosomes (EEA1 endosome marker) and TLR7 (SFig. 4B). Additionally, efficiency of Dotap transfection in BV2 cells was also assessed by monitoring the expression of miR138 by real‐time PCR. As shown in SFig.4C, there was increased expression of miR‐138 in BV2 cells transfected with Dotap‐miR138. Next, we sought to examine whether transfection of BV2 cells with Dotap‐miR138 could result in nuclear translocation of NF‐kB, a downstream mediator of TLR7 pathway. Briefly, BV2 cells were transfected with Dotap‐miR138 mixture for various periods of time (5‐180 min) and assessed for phosphorylation of p65 and translocation of the p65 submit of NF‐kB into the nucleus by western blotting. Transfection of BV‐2 microglia with Dotap‐miR138 (5 pmol/well) resulted in a time‐dependent increase in both phosphorylation of the NF‐kB p65 and translocation of the NF‐kB p65 subunit in the nucleus with a concomitant decrease in its expression in the cytoplasm (SFig. 4D). Additional confirmation of these findings by immunostaining also revealed enhanced translocation of NF‐kB in the nucleus in BV2 cells at 15 min post Dotap‐miR138 exposure (SFig. 4E). To evaluate the role of TLR7 in miR‐138 induced NF‐kB activation, HEK‐Blue‐TLR7 and HEK‐Null cells were exposed to either Dotap‐miR138 or Dotap‐mut‐miR138, followed by assessing the expression of NF‐kB driven reporter SEAP activity. Transfection of BV2 cells with Dotap‐miR138 significantly increased SEAP activity in HEK‐Blue‐TLR7 cells compared to cells loaded with Dotap alone. HEK‐Null cells on the other hand, failed to respond to Dotap‐miR138. In these assays, TLR7 ligand‐ CL264 served as the positive control and significantly increased the SEAP activity in HEK‐Blue‐TLR7 cells, but not in HEK‐Null cells. There was no significant change in SEAP activity of either HEK‐Blue‐TLR7 or HEK‐Null cells transfected with either Dotap‐mut‐miR138 or Dotap‐control (SFig.4F).

**FIGURE 4:**
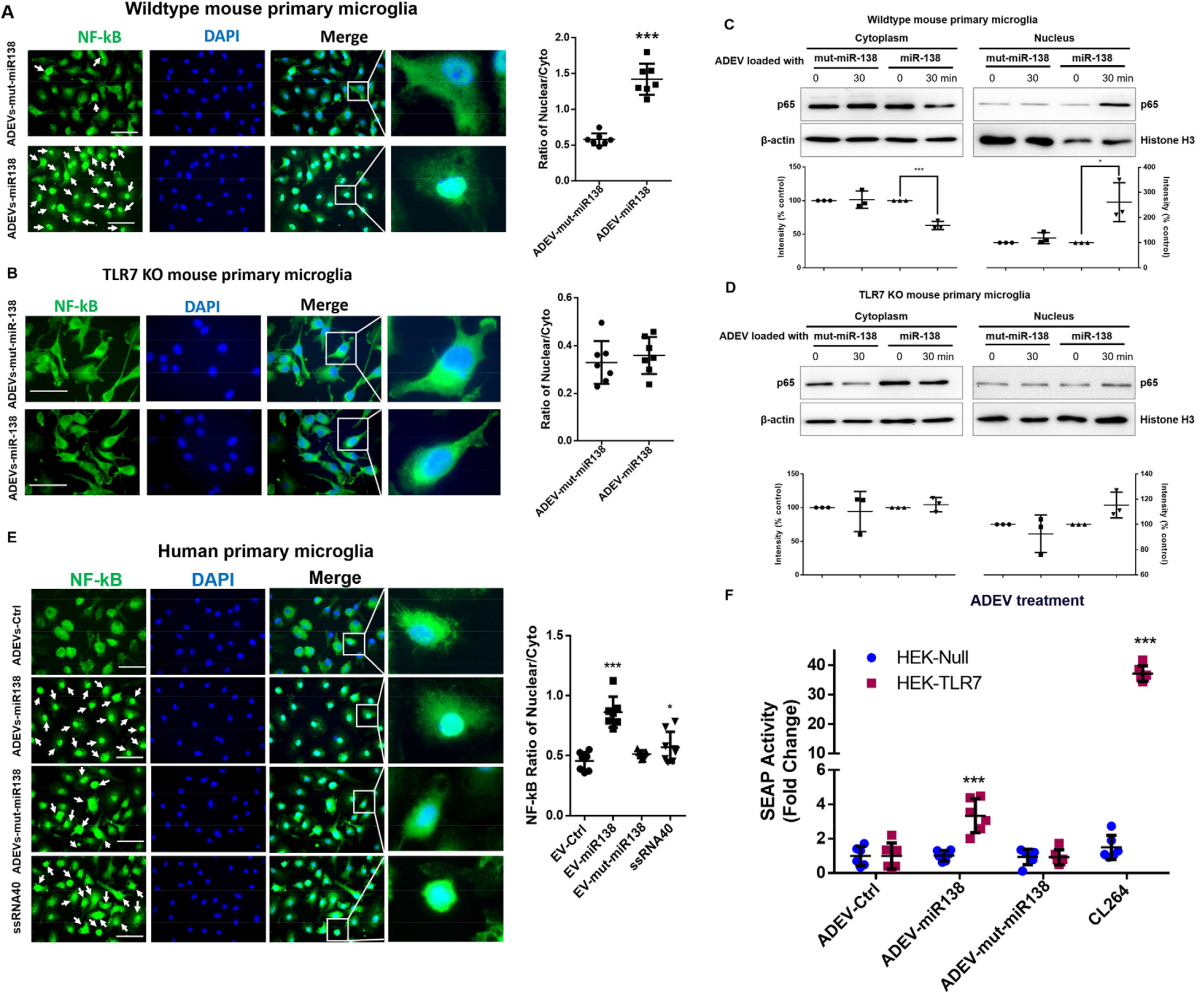
ADEV miR‐138 induces nuclear translocation of NF‐κB p65 involving TLR7. (A) Wild type mouse microglial cells were exposed to ADEVs‐miR‐138 or ADEVs‐mut‐miR‐138, followed by immunostaining with antibody specific for NF‐κB. Bars, 30 μm. White arrows, NF‐κB nuclear translocated cells. The quantification of NF‐κB fluorescence intensity in the nucleus versus non‐ nucleus (*n* = 3, three images were analyzed for each experiment). (B) TLR7 KO mouse microglial cells were exposed to either ADEVs‐miR‐138 or ADEVs‐mut‐miR‐138, followed by immunostaining with antibody specific for NF‐κB. Bars, 10 μm. The quantification of NF‐κB fluorescence intensity in the nucleus versus non‐ nucleus (*n* = 3, three images were analyzed for each experiment). (C) Representative western blot and quantification results of NF‐κB p65 in the lysates of nuclear and cytoplasmic fractions isolated from wild type mouse microglial cells exposed to ADEVs‐miR‐138 or ADEVs‐mut‐miR‐138 for 30 min (*n* = 3). (D) Representative western blot and quantification results of NF‐κB p65 in the nuclear and cytoplasmic fractions isolated from TLR7 KO mouse microglial cells exposed to ADEVs‐miR‐138 or ADEVs‐mut‐miR‐138 for 30 min (*n* = 3). (E) Human primary microglial cells were exposed to ADEVs‐miR‐138, ADEVs‐mut‐miR‐138 or ADEVs‐ssRNA40 (ssRNA40, TLR7 RNA agonist), followed by immunostaining with antibody specific for NF‐κB p65. Bars, 30 μm. White arrows: cells with NF‐κB p65 translocation in the nucleus. The quantification of NF‐κB fluorescence intensity in the nucleus versus non‐ nucleus (*n* = 3, three images were analyzed for each experiment). (F) SEAP activity in HEK‐Null / HEK‐TLR7 cells exposed to ADEVs‐miR‐138 or ADEVs‐mut‐miR‐138 or CL264 (*n* = 3, two wells were analyzed for each experiment). All data are presented as mean ± SD or SEM of three independent experiments. *,*P* < 0.05; **,*P* < 0.01; ***,*P* < 0.001 versus control group.

To examine the effect of ADEV‐miR138 on activation of TLR7 pathway, microglial cells isolated *ex vivo* from wildtype (WT) or TLR7‐knock out (KO) mice were exposed to either ADEV‐miR138 or ADEV‐mut‐miR138 for 30 mins, followed by immunostaining the cells for nuclear translocation of NF‐kB P65 subunit. There was enhanced translocation of NF‐kB P65 into the nucleus of WT microglia exposed to ADEV‐miR138 compared with WT microglia exposed to ADEV‐mut‐miR138 (Figure 4a). Additional confirmation of these findings by immunostaining also revealed enhanced translocation of NF‐kB in the nucleus in human primary microglial cells at 30 min post ADEV‐miR138 or ADEV‐ssRNA40 (ssRNA40: TLR7 RNA agonist) exposure compared with human primary microglia exposed to ADEV‐mut‐miR138 or ADEV‐Ctrl (Figure 4e). Additionally, there were no significant differences in nuclear translocation of NF‐KB P65 in microglia isolated from TLR7‐KO mice exposed to either ADEV‐miR138 or ADEV‐mut‐miR138 (Figure 4b). Further validation of these findings was done by assessing nuclear translocation in the nuclear and cytoplasmic fractions of WT and TLR7 KO microglial cells by western blotting. We first assessed expression of NF‐kB in the nuclear and cytoplasm lysates of mouse primary microglia exposed to ADEV‐miR138 for various time points (0‐3 h). As shown in SFig. 5, ADEV‐miR138 was found to increase the expression of NF‐kB in the nucleus in a time‐dependent manner with a peak expression at 30 min. This was accompanied by a decreased expression in the cytoplasm. We thus chose 30 min time point to compare the effects of ADEV‐miR138 on NF‐kB nuclear translocation in presence of ADEV‐mut‐miR138. As shown in Figure 4c, exposure of WT microglial cells to ADEV‐miR138 (5 pmol/well) resulted in increased translocation of NF‐kB p65 subunit in the nucleus with a concomitant decrease in its expression in the cytoplasm. As expected, there was no significant difference in nuclear translocation of p65 in the microglia isolated from TLR7‐KO mice exposed to either ADEV‐miR138 or ADEV‐mut‐miR138 (Figure 4d). To further evaluate the role of TLR7 in miR‐138 induced NF‐kB activation, HEK‐Blue‐TLR7 and HEK‐Null cells were exposed to either ADEV‐miR138 or ADEV‐mut‐miR138, followed by assessing the NF‐kB driven reporter SEAP activity. Exposure of HEK‐Blue‐TLR7 cells exposed to EV‐miR138 demonstrated a significant increase in SEAP activity compared to the cells exposed to EV‐controls. HEK‐Null cells failed to elicit SEAP activity in presence of either ADEV‐miR138 or ADEV‐mut‐MiR138. In HEK‐Blue‐TLR7 cells, the TLR7 ligand‐CL264 served as the positive control, significantly increasing the SEAP activity. HEK‐Null cells, as expected, failed to elicit any response. There was no significant change in SEAP activity elicited by both HEK‐Blue‐TLR7 or HEK‐Null cells exposed to either ADEV‐Ctrl or ADEV‐mut‐miR138 (Figure 4f).

### 3.5 ADEV miR‐138 activates microglia via TLR7‐NF‐kB signaling pathway

**FIGURE 5:**
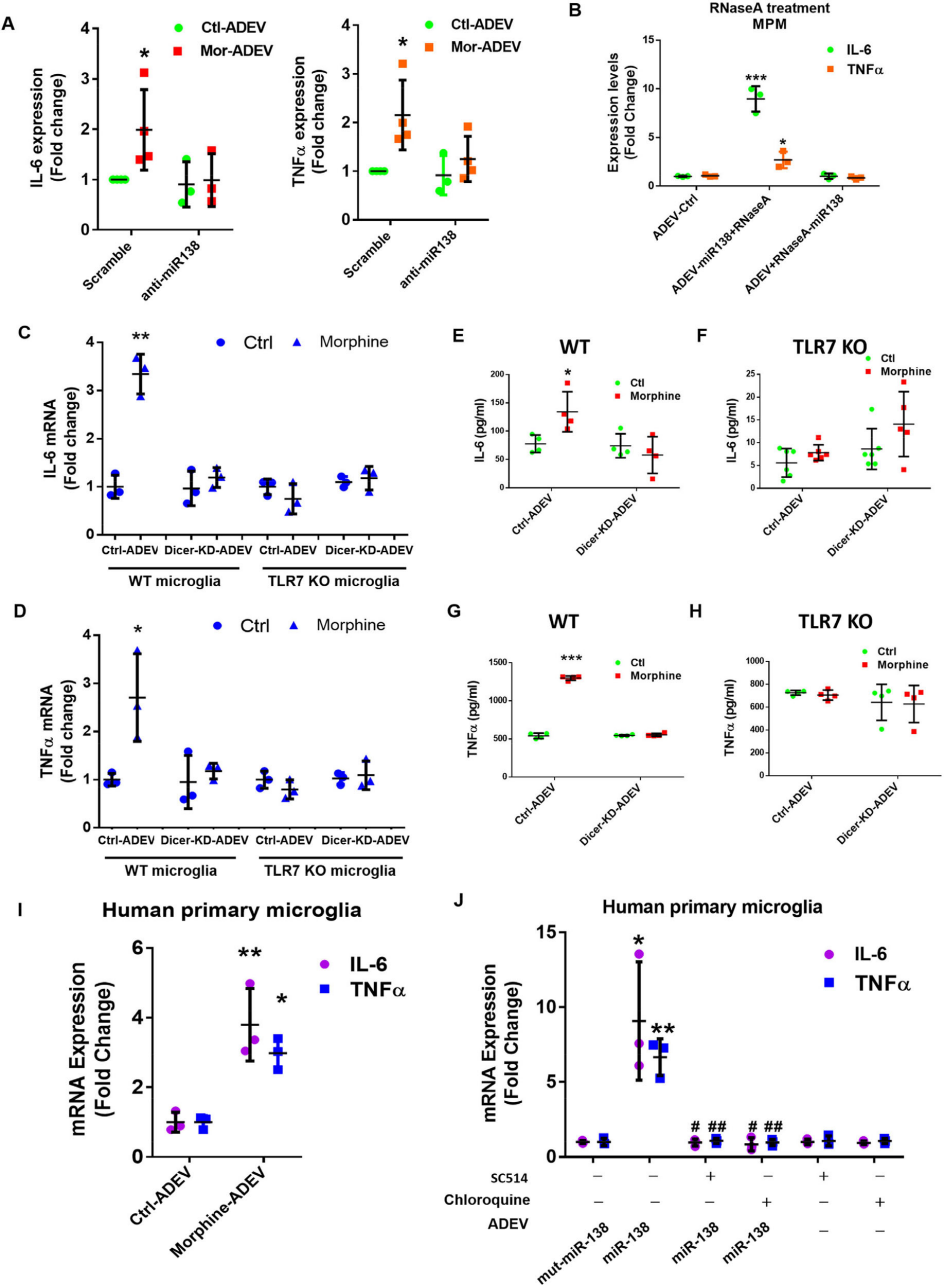
ADEV miR‐138 activates microglia via the TLR7‐NF‐kB signaling pathway. (A) Real‐time PCR analysis of IL‐6 and TNF‐α in mouse primary microglial cells transfected with anti‐miR138 for 24 h followed by exposure to Morphine‐ADEV for additional 4 h (*n* = 4). (B) Real‐time PCR analysis of IL‐6 and TNF‐α in mouse primary microglial cells exposed to ADEV‐miR138+RNaseA (miR138 was first loaded into the ADEV followed by the incubation with RNase) or ADEV +RNaseA+miR138 (miR138 was first incubated with RNase followed by transfection into ADEV) (*n* = 3). (C) Real‐time PCR analysis of IL‐6 in WT/ TLR7 KO microglial cells exposed to Ctrl‐ADEV or morphine‐ADEV isolated from mouse primary astrocytes transfected with Dicer‐siRNA/siRNA‐Ctrl for 24 h, followed by the morphine exposure for an additional 24 h (*n* = 3). (D) Real‐time PCR analysis of TNF‐α in WT/ TLR7 KO microglial cells exposed to Ctrl‐ADEV or morphine‐ADEV isolated from mouse primary astrocytes transfected with Dicer‐siRNA/siRNA‐Ctrl, followed by morphine exposure. IL‐6 was assayed by ELISA in supernatant fluids of WT (E) or TLR7 KO (F) microglial cells cultured for 24 h in the absence or presence of Ctrl‐ADEV or morphine‐ADEV isolated from mouse primary astrocytes transfected with Dicer‐siRNA/siRNA‐Ctrl and followed by morphine exposure (*n* = 4‐6). TNF‐α was assayed by ELISA in the supernatant fluids of WT (G) or TLR7 KO (H) microglial cells cultured for 24 h in the absence or presence of Ctrl‐ADEV or morphine‐ADEV isolated from mouse primary astrocytes transfected with Dicer‐siRNA/siRNA‐Ctrl, followed by the morphine exposure (*n* = 4). (I) Real‐time PCR analysis of IL‐6 / TNF‐α in human primary microglial cells treated with Ctrl‐ADEVs / Morphine‐ADEVs isolated from human primary astrocytes exposed to morphine for 24 h (*n* = 3). (J) Real‐time PCR analysis of IL‐6 / TNF‐α in human primary microglial cells pretreated with endosomal TLR inhibiter Chloroquine, IKK‐2 inhibitor SC514 for 1 h, followed by exposure to ADEVs‐miR‐138 and EVs‐mut‐miR‐138 for additional 4 h (*n* = 3). All data are presented as mean ± SD or SEM of three or four individual experiments. *,*P* < 0.05; **,*P* < 0.01; ***,*P* < 0.001 versus control group. #,*P* < 0.05; ##,*P* < 0.01 versus ADEVs‐miR138 group.

We next sought to determine whether Morphine‐ADEV containing miR138 could induce microglial activation, and if so, whether TLR7‐NF‐kB signaling pathway was involved in this process. BV2 cells were exposed to EVs released from either control or morphine‐stimulated astrocytes (Ctrl‐ADEV / Morphine‐ADEV), followed by assessing the expression of IL‐6 and TNF‐α by real‐time PCR. Exposure of BV2 cells to Morphine‐ADEV resulted in significantly increased expression of IL‐6 and TNF‐α mRNA compared with cells exposed to Ctrl‐ADEVs (SFig. 6A). Here single stranded (ss)RNA40, an HIV‐derived 20‐mer single‐stranded oligoribonucleotide with a GU‐rich sequence, that is a TLR7 agonist, served as a positive control. To validate the role of miR138 in morphine‐ADEVs‐mediated upregulation of IL‐6/TNFα in microglial cells, MPMs were transfected with either anti‐miR138 inhibitor or a scrambled oligo for 24 h, followed by exposure of cells to either morphine‐ADEV or Ctrl‐ADEV and assessed for the expression of IL‐6/TNFα mRNA by real‐time PCR. As shown in Figure 5a, transfection of MPMs with anti‐miR138 ameliorated morphine‐ADEV‐mediated upregulation of IL‐6 and TNFα mRNA.

Next, we sought to examine the direct effect of miR138 on microglial activation. For this BV‐2 cell were transfected with Dotap‐miR138 (5 pmol/well; 4 h) and assessed for the expression of IL‐6 and TNFα mRNAs. Transfection of BV‐2 cells with Dotap‐miR138 resulted in increased mRNA expression levels of IL‐6/TNFα compared to Dotap‐Ctrl. Dotap‐mut‐miR138 failed to induce the expression of IL‐6 and TNFα mRNA levels in BV2 cells (SFig. 6B). Having determined the direct effect of Dotap‐miR138 on cell activation, we next sought to examine the effect of ADEV‐miR138 on activation of microglia. To distinguish the role of EV‐RNA from that of extracellular RNA species not present in the EVs in mediating cellular activation, ADEVs‐miR138 were incubated with RNase (ADEV‐miR138+RNaseA) followed by exposure of MPMs to the ADEVs‐miR138. Cells were then assessed for the expression of IL‐6/TNFα by real‐time PCR. As shown in Figure 5b, ADEV‐miR138 incubated with RNase was able to induce the expression of IL‐6/TNFα in MPMs as expected. Interestingly, EVs wherein miR138 was preincubated with RNase prior to loading into ADEVs (ADEV‐RNaseA‐miR138), failed to induce the expression of IL‐6/TNFα in MPMs.

To examine the specificity of miR‐138 binding to TLR7 leading, in turn, to microglial activation, microglia from WT or TLR7‐KO mice were transfected with Dotap‐miR‐138, Dotap‐scrambled oligo, Dotap‐miR‐196a (lacking the TLR7 binding motif and used here as a negative control) or LPS (positive control) for 4 h and assessed for the expression of IL‐6 and TNF‐α mRNAs. As shown in SFigs. 6C‐D, transfection of WT microglia with Dotap‐miR‐138 or exposure of WT microglia to LPS resulted in significantly increased expression of pro‐inflammatory cytokine mRNAs. Cells transfected with Dotap loaded with an irrelevant miRNA that does not bind to TLR7 such as Dotap‐miR‐196a, failed to induce expression of inflammatory cytokines. Furthermore, microglia from TLR7 KO mice failed to up‐regulate the expression of IL‐6/TNFα, while LPS treatment, as expected, induced the expression of inflammatory cytokines. To further validate whether miR‐138‐mediated induction of IL‐6 and TNFα was regulated translationally, microglia from WT or TLR7‐KO mice were transfected with either Dotap‐miR‐138, Dotap‐scrambled oligo, Dotap‐miR‐196a or LPS for 24 h, followed by assessing supernatant fluids for the expression of IL‐6 and TNFα protein by ELISA. As shown in SFigs. 6E & F, transfection of WT microglia with Dotap‐miR‐138 or LPS resulted in release and up‐regulated expression of inflammatory cytokines while exposure of these cells to Dotap‐miR‐196a failed to exert this effect. In microglia isolated from TLR7 KO mice, Dotap‐miR138 failed to induce up‐regulation of IL‐6 or TNFα.

To further investigate whether morphine‐ADEV‐mediated activation of microglia was attributed to the miRNA cargo in the EVs, mouse primary astrocytes were transfected with either Dicer‐siRNA or scrambled siRNA for 24 h followed by exposure of cells to morphine for additional 24 h. Subsequently, ADEVs were isolated from the conditioned media and incubated with microglia isolated from either WT or TLR7‐KO mice. Four hours later, cell lysates were assessed for the expression of IL‐6 and TNFα by real‐time PCR. ADEVs isolated from Dicer siRNA transfected astrocytes stimulated with morphine failed to induce proinflammatory cytokines compared with ADEVs isolated from morphine‐stimulated astrocytes transfected with scrambled siRNA oligos in microglia from WT mice. In the microglia isolated from TLR7 KO mice, on the other hand, morphine‐ADEVs isolated from either Dicer‐siRNA or scrambled‐siRNA transfected astrocytes failed to induce the expression of IL‐6 or TNFα mRNA (Figure 5c and d).

To confirm whether increased expression of IL‐6 and TNFα mRNA also translated into increased protein expression of these cytokines, parallel studies involving assessment of supernatant fluids for the expression of cytokines by ELISA were also carried out. Similar to the real‐time PCR results, morphine‐ADEVs isolated from siRNA‐Ctrl transfected astrocytes induced the expression of IL‐6/ TNFα mRNA in WT microglia compared with WT microglia exposed to Ctrl‐ADEVs. Morphine‐ADEV isolated from siRNA Dicer transfected astrocytes on the other hand, failed to induce the expression of IL‐6/ TNFα proteins in WT microglia (Figure 5e and g). Furthermore, morphine‐ADEVs isolated from siRNA‐Ctrl transfected astrocytes failed to induce IL‐6/ TNFα protein levels in microglia isolated form TLR7‐KO mice (Figure 5f and h).

We next validated our findings in human primary astrocytes and microglial cells. Human primary microglial cells were exposed to ADEVs isolated from either morphine stimulated or control human primary astrocytes and assessed for the expression of IL‐6 and TNFα mRNA by real‐time PCR. As shown in Figure 5i, there was increased expression of IL‐6 and TNFα mRNA in human primary microglia exposed to morphine‐ADEVs compared with ADEVs isolated astrocytes not exposed to morphine. To validate whether morphine‐ADEV mediated upregulation of IL‐6 and TNFα mRNA involved miR‐138‐TLR7‐NF‐kB signaling axis, human primary microglial cells were pretreated with either chloroquine (TLR7 inhibitor) or SC‐514 (NF‐kB inhibitor) followed by transfection of cells with Dotap‐miR‐138. As expected, cells transfected with Dotap‐miR138 demonstrated induced expression of IL‐6 and TNFα mRNA, and furthermore, pretreatment of cells with either chloroquine or SC‐514 abrogated miR‐Dotap‐miR‐138‐mediated up‐regulation of the cytokine mRNAs (SFig. 6G). These studies were also performed using ADEV‐miR‐138 and as shown in Figure 5j, and similar to Dotap‐miR‐138, ADEV‐miR‐138 also induced activation of microglia which was significantly inhibited by both chloroquine and SC‐514.

### 3.6 Morphine‐induced microglial activation involves TLR7 in vivo

**FIGURE 6:**
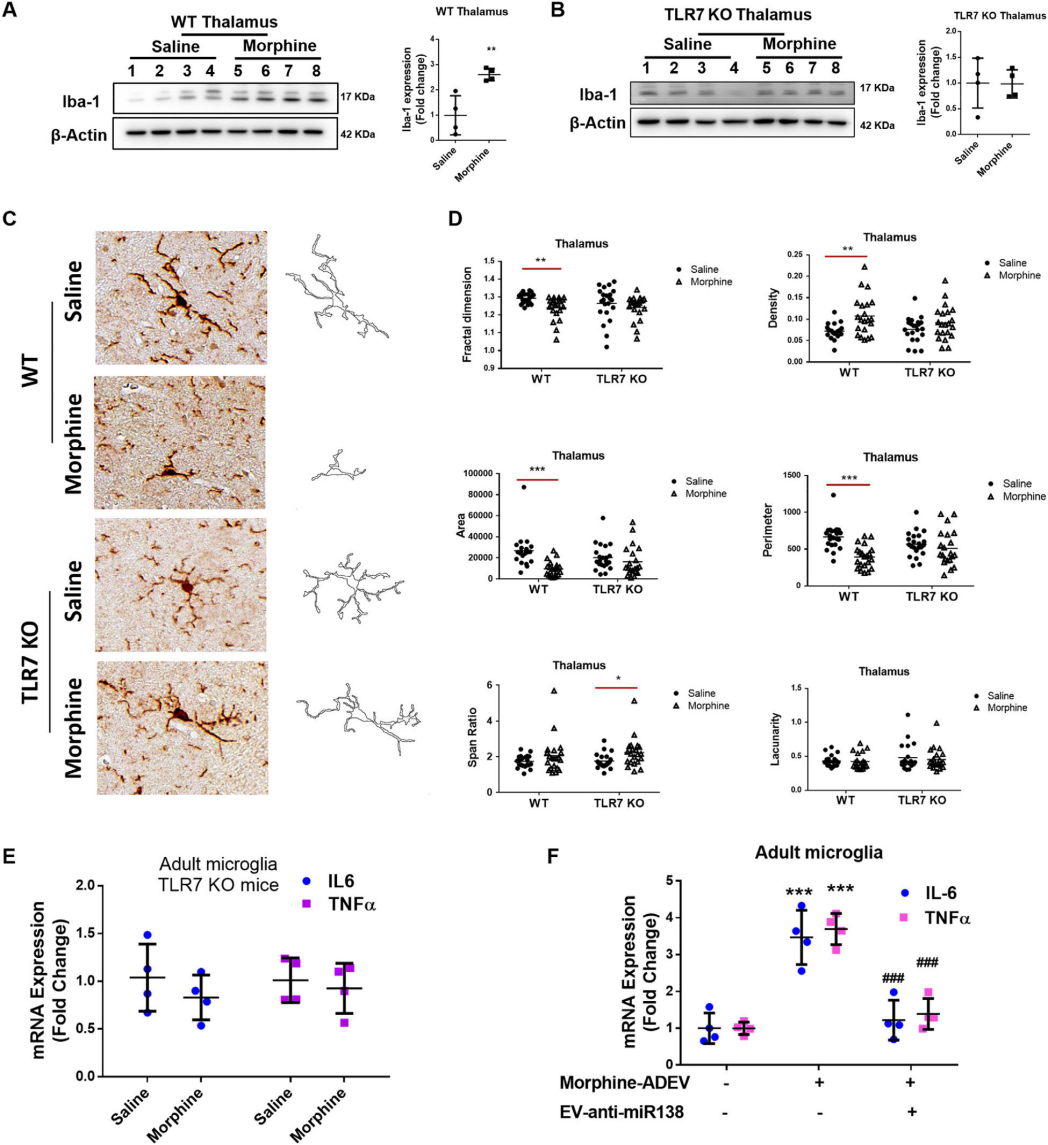
Morphine‐induced microglial activation involves activation of TLR7. (A) Representative western blot and quantification of Iba1 in the lysates from the thalamus of WT mice administrated saline or morphine (*n* = 4/group). (B) Representative western blot and quantification of Iba1 in the thalamus lysates of TLR7 KO mice administrated saline or morphine (*n* = 4/group). (C) Representative images of Iba‐1+ cells and pairwise outline shapes were used for morphological parameters measures in the thalamus of WT or TLR7 KO mice administrated saline or morphine. (D) Quantification of morphological parameters: fractal dimension, density, area, perimeter, span ratio and lacunarity in the thalamus of WT and TLR7 KO mice administrated saline or morphine (5 or 6 cells/animal). (E) Real‐time PCR analysis of IL‐6 /TNFα mRNA expression in adult microglial cells isolated from the brains of TLR7 KO mice administrated morphine (*n* = 4). (F) Real‐time PCR analysis of IL‐6 /TNFα mRNA expression in adult microglial cells isolated from the brains of mice administrated EV‐anti‐miR138 followed by Ctrl‐ADEV/Morphine‐ADEVs (*n* = 4). All data are presented as mean ± SD or SEM of four independent experiments. *,*P* < 0.05; **,*P* < 0.01; ***,*P* < 0.001 versus saline group, ###,*P* < 0.001 versus morphine‐ADEV group.

Based on the fact that there was significant increase in expression of Iba‐1 coupled with morphological changes in the microglia primarily in the thalamus region of morphine‐administered C57BL/6N mice, and since TLR7 played a critical role in microglial activation in response to morphine‐ADEVs, we next sought to determine the role of TLR7 in increased microglial activation and also to determine microglial morphological changes in the thalamus of morphine‐ administered mice. For this WT and TLR7‐KO mice were injected with morphine three times a day for 7 consecutive days, followed by assessing the expression of microglial marker Iba1 in the thalamus by western blotting. In parallel, microglial morphological changes in the thalamus were also examined by immunostaining with Iba1. As shown in Figure 6a, Iba‐1 expression was significantly upregulated in the thalamus (Figure 6a) of WT mice versus TLR7‐KO mice administrated morphine. Saline administered WT and TLR7 KO animals failed to demonstrate upregulation of Iba‐1 (Figure 6b). For examining microglial morphological changes, in Figure 6c and d we evaluated the same parameters as described earlier in Figure 1b and c. The fractal dimension, area, and perimeter of microglia were significantly decreased in the thalamus of WT mice administrated morphine. There were no significant differences in these parameters in the thalamus of TLR7‐KO mice administrated either morphine or saline. Density of Iba‐1+ cells were found to be significantly increased in the thalamus of WT mice administrated morphine, In contrast there was no change in the density in TLR7‐KO mice administrated either morphine or saline (Figure 6d).

Further validation of the role of TLR7 in morphine‐mediated activation of microglia involved isolating adult microglial cells ex vivo from TLR7 KO mice administrated either saline or morphine followed by sorting the cells by labeling with CD11b and CD45 antibodies using flow cytometry. Enriched CD11b^high^CD45^low^ microglial cells were assessed for the expression of IL‐6 and TNFα mRNA by real‐time PCR. As shown in Figure 6e, morphine failed to induce the expression of IL‐6 or TNFα ex vivo adult microglia isolated from TLR7 KO mice.

Next step was to examine the involvement of ADEV‐miR138 in morphine‐mediated activation of microglia in vivo. For this one group of WT mice were intranasally administered either Ctrl‐ADEVs or morphine‐ADEVs, and another group of mice were pretreated daily intranasally with ADEVs loaded with anti‐miR138 1 h prior to intranasal delivery of morphine‐ADEVs for 7 consecutive days. One hour after the last intranasal delivery of Ctrl‐ADVEs or morphine‐ADEVs on day 7, mice were sacrificed followed by isolation of adult microglia that were subsequently assessed for the expression of IL‐6 and TNFα by real‐time PCR. As shown in Figure 6f, and as expected, expression levels of both IL‐6 and TNFα were found to be significantly increased in the ex vivo adult microglia isolated from mice treated with morphine‐ADEVs compared with animals treated with Ctrl‐ADEVs. In contrast, microglia isolated from morphine‐ADEVs administered mice treated with ADEV‐anti‐miR138 failed to demonstrate induced expression of proinflammatory cytokines.

## 4 DISCUSSION

Morphine has been reported to accelerate progression of HAND involving activation of astrocytes and microglia and also enhance neuronal injury in the CNS (Dutta et al., 2012; El‐Hage et al., 2008; Fernández‐Arjona et al., 2017; Fitting et al., 2014; Gurwell et al., 2001; Hahn et al., 2016) EV‐associated miRNAs, the well‐studied constituents of the EV cargo, are known to play critical roles in cell‐to‐cell communication, leading to impairment of cellular functions. For example, in our earlier study, we have shown that both morphine and HIV Trans‐Activator of Transcription (Tat) protein upregulated the expression of miR‐29b in ADEVs, which, in turn, was taken up by neurons leading to neuronal apoptosis (Hu et al., 2012). Along these lines, studies by Yelamanchili et al. have also demonstrated that HIV‐1 infection of blood‐derived macrophages led to significant upregulation of miR‐21, which upon uptake by the neurons resulted in activation of the TLR7‐dependent necroptosis pathway (Yelamanchili et al., 2015). TLRs that are expressed in most cell types of the CNS, play a crucial role in the initiation of host immune responses following exposure to noxious stimuli, including but not limited to, HIV‐1 infection (Hernández, Stevenson, Latz, & Urcuqui‐Inchima, 2012), Alzheimer's Disease (Gambuzza et al., 2014) and Parkinson's Disease (Dzamko et al., 2017). Role of TLR signaling has also been reported in morphine‐mediated activation of microglia, likely contributing to increased progression of neuropathogenesis in HAND commonly observed in HIV‐infected opioids abusers (Dutta et al., 2012). In our previous findings we demonstrated that EVs derived from morphine‐stimulated astrocytes could be taken up by microglia, leading, in turn, activation of the TLR7 signaling pathway, ultimately impacting impairment of microglial phagocytosis (Hu et al., 2018). In the current study, for the first time we demonstrate that upregulation of miR138 in morphine‐stimulated astrocyte EVs can be shuttled into microglia, resulting in activation of these latter cells. In agreement with the in vitro results, our in vivo data also demonstrated increased activation of microglia that was accompanied with morphological alterations in the thalamus of morphine‐administered mice.

It has been well documented that microglial morphology and functions are closely related (Fernández‐Arjona et al., 2017; Fernández‐Arjona et al., 2019), however, most studies are often focused on proinflammatory cytokine release and Iba1 expression as indices of microglial activation; alterations in microglial morphology are often reported separately and not in conjunction with these indices (Bowyer et al., 2020; Liu et al., 2020; Zhang, Zhang, & You, 2018). In the present study we examined the expression of microglial marker Iba1 in various brain regions of WT or TLR7 KO mice administered either morphine or saline. Expression of microglial activation marker, Iba‐1 was found to be significantly upregulated preferentially in the thalamus of WT mice administered morphine compared to saline controls. There were no significant differences in Iba‐1 expression in TLR7‐KO mice administered either morphine or saline. Besides the Iba‐1 expression levels, we also examined a set of six key morphological parameters to evaluate changes in microglial morphology in response to morphine. These included, fractal dimension, density, area, perimeter, span ratio, lacunarity. Four of the six morphological parameters (fractal dimension, density, area, and perimeter) were found to be significantly altered in the thalamus of WT mice administered morphine compared to saline controls. As expected, this phenomenon was not observed in TLR7 KO mice administered morphine. These findings are in agreement with earlier reports describing thalamus as the preferred region of morphine action in the CNS (Emmers, 1984; Hutchinson et al., 2009; Lee et al., 2018; Matzeu, Zamora‐Martinez, & Martin‐Fardon, 2014). There are several reports implicating by in situ hybridization and immunohistochemical location that thalamus has the highest distribution and density of the mu‐opioid receptor (Herkenham & Pert, 1982; Sim, Selley, & Childers, 1995; Wang et al., 1993), which likely could explain the microglial morphological changes preferentially observed in this region in our current study. We examined the proinflammatory cytokine IL6 / TNFα mRNA levels in isolated adult microglia from brains of mice administered either morphine or saline, as an indicator of microglial activation (Wang, Tan, Yu, & Tan, 2015). TNFα and IL6 are considered as the major proinflammatory cytokines induced by the TLR agonist (Wu et al., 2015), and, have been reported to be upregulated via activation of TLR7 (Mcnitt & Lukefahr, 1990). We found that morphine induced the expression of IL‐6 or TNFα in ex vivo adult microglia isolated from WT mice, but not TLR7 KO mice.

MicroRNAs (miRNAs) have emerged as key regulators of gene regulation controlling almost every cellular process (Ambros, 2003; Bartel, 2004). MiRNAs control gene expression at the post‐transcription level by regulating degradation of target mRNAs or by regulating translational repression (Bartel, 2009; Filipowicz, Bhattacharyya, & Sonenberg, 2008; Xiang et al., 2019). Intriguingly, single‐stranded RNAs have also been shown to activate innate cellular TLRs leading to generation of inflammatory cytokines and activation of NF‐kB pathway, critical for mediating inflammatory responses (Zhang et al., 2016). It has been previously reported that certain miRNAs contain “immunostimulatory” G/U rich sequences; specifically, uridine clusters that define the innate stimulatory activity of RNA (Heil, 2004). These G/U rich sequences can bind to TLRs and specifically activate TLR7 in mice (Fabbri et al., 2012) & TLR8 in humans (Akira, Takeda, & Kaisho, 2001). Several miRNAs carrying these specific motifs have been shown to activate the TLR signaling pathways (Fabbri et al., 2012; Lehmann et al., 2012; Yelamanchili et al., 2015). For example, a report identified extracellular miR let‐7, containing a TLR7 binding motif, was shown to be a potent activator of TLR7 signaling in macrophages & microglia (Lehmann et al., 2012). In addition, miR‐21 and miR‐29a also have been demonstrated to directly bind to TLR7/8 resulting in release of proinflammatory cytokines in macrophages (Fabbri et al., 2012). Similar to this study, our findings also demonstrate that miR‐138 released in morphine‐stimulated astrocyte EVs, was taken up by the microglia, resulting in activation of TLR7/8 signaling pathway via the interaction of miR138 withTLR7. It must be noted that miR‐138 is a GU‐rich single‐stranded RNA with a high potential to bind with TLR7.

Having found the enrichment of miR‐138 in morphine‐ADEVs, and its activation of microglia via the TLR7 signaling pathway, we next sought to examine the binding of miR‐138 to endosomal TLR7 by RNA immunoprecipitation (RIP) assay. We found miR‐138 highly enriched in the TLR7 co‐immunoprecipitated complex compared with the IgG control. This phenomenon was also validated in the HEK‐TLR7 cells, wherein we found enrichment of miR‐138 in the TLR7 co‐immunoprecipitated complex in HEK‐TLR7 cells compared with the HEK‐Null cells. Furthermore, we also validated the interaction of miR138 and TLR7 by pull down assay using biotin‐labelled miR138. There was enrichment of TLR7 in miR‐138‐biotin complex compared with lysates from miR‐mut‐138‐biotin transfected cells. These findings thus confirm the ability of EV‐miR‐138 to activate microglia via interaction and activation of the TLR pathway. It must be pointed out though that our studies do not explain how miR‐138 is transported to the endosomes and remains an area of future investigations.

Our findings offer novel insights into the key function of EV miRNAs in mediating neuroinflammation, underscoring a critical role of miR‐138 released from morphine‐stimulated astrocytes. Besides miR138, other EV‐miRNAs are also implicated in the onset of neuroinflammation in various neuroinflammatory response. For example, Francesc Ibáñez et.al demonstrated that during ethanol exposure, dysregulation of ADEV‐miRNAs such as miR‐146a, miR‐182 and miR‐200b resulted in apoptotic cell death in neurons, leading, in turn, to an amplified neuroinflammatory response (Ibáñez, Montesinos, Ureña‐Peralta, Guerri, & Pascual, 2019). Furthermore, ADEV‐miRNAs released from activated astrocytes have also been implicated as potential neuroinflammatory biomarkers in traumatic brain injury (TBI) (Gayen, Bhomia, Balakathiresan, & Knollmann‐Ritschel, 2020). Additionally, in a mouse model of TBI, increased levels of miRNA‐21 have been reported in EVs compared with the EVs from control mice, and this, in turn, was suggested to induce microglial activation with implications for the pathophysiological responses observed in TBI (Harrison et al., 2016). Our findings point to the activation of innate cellular receptors by miRNAs and is in line with yet another mechanism by which the EV cargoes can impact the functions of recipient cells. This mechanism highlights the intricate pathways used by donor cells to affect functioning of the distant neighboring cells. In our previous study we found 14 additional EV‐miRNAs besides miR138, that contained the TLR7/8 binding motifs and that were significantly upregulated in morphine‐ADEVs compared with control‐ADEVs. All these EV‐miRNAs could activate TLR7 and contribute to morphine‐ADEV‐mediated microglial activation. Interestingly, miR138 was also found to be upregulated in the basal ganglia of SIV‐infected rhesus macaques compared with the uninfected controls using miRNA array analysis, in our previous study, thereby suggesting that miR138 could also play role in SIV infection‐mediated neuroinflammation (Hu et al., 2019). The underlying mechanism(s) as well as the role of ADEV‐miR138 in the process warrants further investigation.

Functional implications of morphine‐mediated activation of microglia involving morphine‐ADEV and its reversal was also validated using an in vivo model. For this wild type mice were intranasally delivered either Ctrl‐ADEVs or morphine‐ADEVs, and another group of mice pretreated with intranasally delivered ADEVs loaded with anti‐miR138 prior to intranasal delivery of mice with morphine‐ADEVs daily, for 7 consecutive days. Our findings demonstrated that intranasal delivery of morphine‐ADEVs in WT mice resulted in microglial activation and furthermore, that pretreatment of mice with intranasal delivery of ADEV‐loaded anti‐miR138 resulted in restoration of morphine‐ADEV mediated microglial activation. These findings are in agreement with the recent studies implicating the role of EV miRNAs as mediators of cellular activation  (Bian et al., 2019; Chaudhuri et al., 2018; Vu et al., 2019).

**FIGURE 7:**
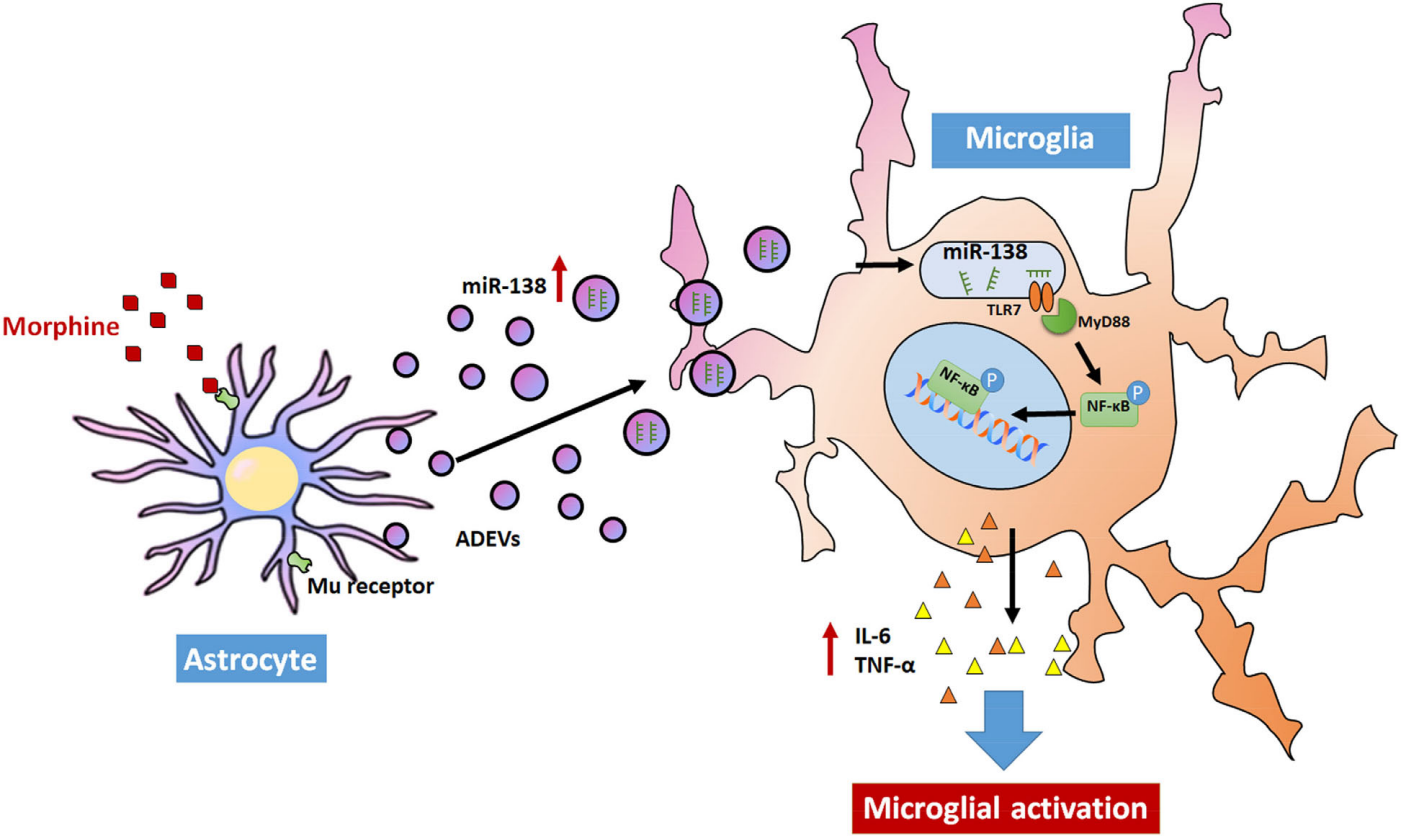
Schematic diagram demonstrating signaling pathways involved in Morphine‐ADEV‐mediated microglial activation. Morphine induces the expression of miR138 in morphine‐stimulated astrocyte‐derived EVs, which can be taken up by microglial cells. Morphine‐ADEVs containing miR138, in turn, activates the TLR7‐NF‐kB axis and induces the expression and release of IL6 / TNFα, ultimately leading to microglial activation.

In summary, our findings demonstrate a novel molecular pathway of morphine‐mediated induction of microglial activation, involving release of miRNA and uptake of EV by microglia and activation of endosomal TLR7 (Figure 7). These findings could have implications for HIV‐1‐infected heroin abusers that are known to have increased risk of neuroinflammation and can also have ramifications for the development of EV‐loaded RNA‐based therapeutics for the treatment of neuroinflammation in opioids abusers. These findings could also have applications for treatment of individuals who are on chronic pain killers such as cancer patients or the elderly with susceptibility to neurodegenerative diseases where morphine could exacerbate neuroinflammation and worsen disease pathogenesis. While the role of morphine in peripheral pathogenesis is well studied (Kim et al., 2016; Vasko, Henney, Oldham, Brawley, & Morrow, 1966; Wang et al., 2018; Zhang et al., 2019), highlighting its role in tissues such as the CNS, is of prime importance and warrants further investigations.

## CONFLICT OF INTEREST

The authors declare no competing financial interests in relation to the work described.

## Supporting information


**SFigure. 1 Morphine‐induced microglial activation**. (A) Representative western blot and quantification of Iba1 in the lysates of cortices of mice administrated saline or morphine (n = 4/group). (B) Representative images of Iba‐1+ cells and pairwise outline shapes were used for morphological parameters measures in the cortices of mice administrated saline or morphine. (C) Quantification of morphological parameters fractal dimension, density, area, perimeter, span ratio, and lacunarity in cortices of mice administrated saline or morphine (n = 5 or 6/animal). (D) Representative western blot and quantification of Iba1 in the hippocampus lysates of mice administrated saline or morphine. (E) Representative images of Iba‐1+ cells and pairwise outline shapes were used for morphological parameters measures in the hippocampi of mice administrated saline or morphine. (F) Quantification of morphological parameters: fractal dimension, density, area, perimeter, span ratio and lacunarity in the hippocampi of mice administrated saline or morphine (n = 5 or 6/animal). All data are presented as mean ± SD or SEM of four individual experiments. *, *p* < 0.05; **, *p* < 0.01; ***, *p* < 0.001 versus saline group using Student's t test.

**SFigure 2. Characterization of EVs Isolated from Astrocyte Cultures**. (A) Western blot characterization of mouse primary astrocyte EVs using the exosome marker antibodies specific for TSG101, CD63, and Alix. Calnexin was used as a control for cell debris contamination. (B) Size and particle distribution plots of isolated EVs from mouse primary astrocyte by ZetaView. The plot shows a peak size around 100 nm for the isolated EVs. (C) Western blot characterization of A172 astrocyte EVs with exosome marker antibodies specific for TSG101, CD63, and Alix. Calnexin was used as a control for cell debris contamination. (D) Size and particle distribution plots of isolated EVs from A172 astrocyte by ZetaView. The plot shows a peak size around 100 nm for the isolated EVs. (E) Western blot characterization of human primary astrocyte EVs with exosome marker antibodies specific for TSG101, CD63, and Alix. Calnexin was used as a control for cell debris contamination. (F) Size and particle distribution plots of isolated EVs from human primary astrocyte by ZetaView. The plot shows a peak size around 100 nm for the isolated EVs. (G) AFM image of EVs isolated from mouse primary astrocyte culture supernatants. All experiments were done for at least three independent times.

**SFigure 3. EVs are internalized by the microglia and reach the endosomes**. (A & B) Representative fluorescence images of mouse primary microglial cells incubated with EVs purified from pEF6.mCherry‐TSG101‐transfected mouse primary astrocytes for 30 min, followed by immunostaining of microglial marker Iba‐1 (A) and the early endosome marker (EEA1) (B). Iba1 (Green), EEA1(Green), EV‐TSG101‐mCherry (Red). Bars, 30 μm (n = 3). (C) Representative fluorescence images of mouse primary microglial cells incubated with ADEV‐Cy5‐miR138+RNaseA (Cy5‐miR138 was first loaded into the ADEV followed by the incubation with RNase) or ADEV+RNaseA‐Cy5‐miR138 (Cy5‐miR138 was first incubated with RNase followed by loaded into ADEVs) followed by immunostaining MPMs for the endosomal marker EEA1. Bars, 30 μm (n = 3).

**SFigure 4. Dotap‐miR138 mediated NF‐kb nuclear translocation in BV2 cells. (A)** Representative fluorescence images of BV2 cells transfected with DOTAP‐Cy5‐miR138 for 30 min, followed by the immunostaining of (A) microglial marker Iba1 (Green), Bars, 20 μm, (B) early endosome marker EEA1 (Green) and TLR7 (Green). Bars, 5 μm (n = 3). (C) Real‐time PCR analysis of miR138 mRNA expression in BV2 cells transfected with various concentration of DOTAP‐miR138 (2.5, 5, 10 pmol / well) (n = 3). (D) Representative western blot of p‐NF‐κB p65 in the lysates or NF‐κB p65 in the nuclear and cytoplasmic lysates isolated from BV2 cells transfected with DOTAP‐miR‐138 for various time points (5 min to 3 h) (n = 3). (E) BV2 cells were transfected with DOTAP‐miR‐138 or DOTAP‐mut‐miR‐138, followed by immunostaining with antibodies specific for NF‐κB p65. Bars, 30 μm. White arrows, NF‐κB nuclear translocated cells (n = 3, two images were analyzed each experiment). (F) SEAP activity in HEK‐Null / HEK‐TLR7 cells exposed to DOTAP‐miR‐138 or DOTAP‐mut‐miR‐138 or CL264 (n = 3, two wells were tested each experiment). All data are presented as mean ± SD or SEM of three individual experiments. **, *p* < 0.01; ***, *p* < 0.001 versus control group.

**SFigure 5. ADEV‐miR138 mediated NF‐kb (p65) nuclear translocation in MPMs**. (A, B) Representative western blot of NF‐κB p65 in the cytoplasmic (A) and nuclear lysates (B) isolated from MPMs exposed to ADEV‐miR‐138 for various time points (5 min to 3 h). All data are presented as mean ± SD or SEM of three individual experiments. *, p < 0.05; **, p < 0.01; ***, p < 0.001 versus control group.

**SFigure 6. DOTAP miR‐138 activates microglia via the TLR7‐NF‐kB signaling pathway**. (A) Real‐time PCR analysis of IL‐6 and TNF‐α in BV‐2 cells treated with EVs isolated from control or morphine stimulated astrocytes or ssRNA40. (B) Real time PCR analysis of IL‐6 and TNF‐α in BV‐2 cells transfected with DOTAP formulations of miR‐138 or mutant‐miR‐138. Real‐time PCR analysis of IL‐6 (C) and TNFα (D) in WT or TLR7 KO microglial cells transfected with Dotap‐miR138, DOTAP‐miR196 or LPS. IL‐6 (E) and TNFα (F) was assayed by ELISA in supernatants of WT or TLR7 KO microglial cells transfected with or without Dotap‐miR138, DOTAP‐miR196, or treated with LPS. (G) Real time PCR analysis of IL‐6 and TNF‐α in human primary microglial cells pretreated with endosomal TLR inhibiter Chloroquine, IKK‐2 inhibitor SC514 for 1 hr, followed by exposure to DOTAP‐miR‐138 and DOTAP‐mut‐miR‐138 for additional 4 hr. All data are presented as mean ± SD or SEM of three independent experiments. *, *p* < 0.05; **, *p* < 0.01; ***, *p* < 0.001 versus control group.

## References

[jev212027-bib-0001] Akira, S. , Takeda, K. , & Kaisho, T. (2001). Toll‐like receptors: Critical proteins linking innate and acquired immunity. Nature Immunology, 2(8), 675–680. 11477402 10.1038/90609

[jev212027-bib-0002] Alam, A. (2012). Long‐term analgesic use after low‐risk surgery: A retrospective cohort study. Archives of Internal Medicine, 172(5), 425–430. 22412106 10.1001/archinternmed.2011.1827

[jev212027-bib-0003] Ambros, V. (2003). MicroRNA pathways in flies and worms: Growth, death, fat, stress, and timing. Cell, 113(6), 673–676. 12809598 10.1016/s0092-8674(03)00428-8

[jev212027-bib-0004] Bartel, D. P. (2009). MicroRNAs: Target recognition and regulatory functions. Cell, 136(2), 215–233. 19167326 10.1016/j.cell.2009.01.002 PMC3794896

[jev212027-bib-0005] Bartel, D. P. (2004). MicroRNAs: Genomics, biogenesis, mechanism, and function. Cell, 116(2), 281–297. 14744438 10.1016/s0092-8674(04)00045-5

[jev212027-bib-0006] Bian, E. B. , Chen, E. F. , Xu, Y. D. , Yang, Z. H. , Tang, F. , Ma, C. C. , … Zhao, B. (2019). Exosomal lncRNAATB activates astrocytes that promote glioma cell invasion. International Journal of Oncology, 54(2), 713–721. 30483768 10.3892/ijo.2018.4644

[jev212027-bib-0007] Bowyer, J. F. , Sarkar, S. , Burks, S. M. , Hess, J. N. , Tolani, S. , O'callaghan, J. P. , & Hanig, J. P. (2020). Microglial activation and responses to vasculature that result from an acute LPS exposure. Neurotoxicology, 77, 181–192. 32014511 10.1016/j.neuro.2020.01.014 PMC7294247

[jev212027-bib-0008] Cai, Yu , Yang, Lu , Hu, G. , Chen, X. , Niu, F. , Yuan, Li , … Buch, S. (2016). Regulation of morphine‐induced synaptic alterations: Role of oxidative stress, ER stress, and autophagy. Journal of Cell Biology, 215(2), 245–258. 10.1083/jcb.201605065 PMC5084649 27810915

[jev212027-bib-0009] Caruso Bavisotto, C. , Scalia, F. , Marino Gammazza, A. , Carlisi, D. , Bucchieri, F. , Conway De Macario, E. , … Campanella, C. (2019). Extracellular vesicle‐mediated cell(‐)cell communication in the nervous system: Focus on neurological diseases. International Journal of Molecular Sciences, 20(2), 434 10.3390/ijms20020434 PMC6359416 30669512

[jev212027-bib-0010] Casamayor, M. , Didonato, K. , Hennebert, M. , Brazzi, L. , & Prosen, G. (2018). Administration of intravenous morphine for acute pain in the emergency department inflicts an economic burden in Europe. Drugs Context, 7, 1.

[jev212027-bib-0011] CDC (2018) U.S. Opioid Prescribing Rate Maps. (CDC).

[jev212027-bib-0012] Chaudhuri, A. D. , Dastgheyb, R. M. , Yoo, S.‐W. , Trout, A. , Talbot Jr, C. C. , Hao, H. , … Haughey, N. J. (2018). TNFalpha and IL‐1beta modify the miRNA cargo of astrocyte shed extracellular vesicles to regulate neurotrophic signaling in neurons. Cell death & disease, 9(3), 363. 29507357 10.1038/s41419-018-0369-4 PMC5838212

[jev212027-bib-0013] Dave, R. S. (2012). Morphine affects HIV‐induced inflammatory response without influencing viral replication in human monocyte‐derived macrophages. Fems Immunology and Medical Microbiology, 64(2), 228–236. 22066570 10.1111/j.1574-695X.2011.00894.x PMC3336009

[jev212027-bib-0014] Devhare, P. B. , & Ray, R. B. (2018). Extracellular vesicles: Novel mediator for cell to cell communications in liver pathogenesis. Molecular Aspects of Medicine, 60, 115–122. 29122679 10.1016/j.mam.2017.11.001 PMC5856598

[jev212027-bib-0015] Dutta, R. , Krishnan, A. , Meng, J. , Das, S. , Ma, J. , Banerjee, S. , … Roy, S. (2012). Morphine modulation of toll‐like receptors in microglial cells potentiates neuropathogenesis in a HIV‐1 model of coinfection with pneumococcal pneumoniae. Journal of Neuroscience, 32(29), 9917–9930. 22815507 10.1523/JNEUROSCI.0870-12.2012 PMC3417042

[jev212027-bib-0016] Dutta, R. , & Roy, S. (2012). Mechanism(s) involved in opioid drug abuse modulation of HAND. Current HIV Research, 10(5), 469–477. 22591371 10.2174/157016212802138805 PMC3565579

[jev212027-bib-0017] Dzamko, N. , Gysbers, A. , Perera, G. , Bahar, A. , Shankar, A. , Gao, J. , … Halliday, G. M. (2017). Toll‐like receptor 2 is increased in neurons in Parkinson's disease brain and may contribute to alpha‐synuclein pathology. Acta Neuropathologica, 133(2), 303–319. 27888296 10.1007/s00401-016-1648-8 PMC5250664

[jev212027-bib-0018] El‐Hage, N. , Bruce‐Keller, A. J. , Yakovleva, T. , Bazov, I. , Bakalkin, G. , Knapp, P. E. , & Hauser, K. F. (2008). Morphine exacerbates HIV‐1 Tat‐induced cytokine production in astrocytes through convergent effects on [Ca(2+)](i), NF‐kappaB trafficking and transcription. Plos One, 3(12), e4093. 19116667 10.1371/journal.pone.0004093 PMC2605563

[jev212027-bib-0019] El‐Hage, N. , Rodriguez, M. , Dever, S. M. , Masvekar, R. R. , Gewirtz, D. A. , & Shacka, J. J. (2015). HIV‐1 and morphine regulation of autophagy in microglia: Limited interactions in the context of HIV‐1 infection and opioid abuse. Journal of Virology, 89(2), 1024–1035. 25355898 10.1128/JVI.02022-14 PMC4300622

[jev212027-bib-0020] Emmers, R. (1984). Changes in thalamic nociception resulting from morphine‐ and meperidine‐dependence in rats. Experimental Neurology, 83(1), 118–133. 6537810 10.1016/0014-4886(84)90051-7

[jev212027-bib-0021] Eylem, C. C. , Yilmaz, M. , Derkus, B. , Nemutlu, E. , Camci, C. B. , Yilmaz, E. , … Emregul, E. (2020). Untargeted multi‐omic analysis of colorectal cancer‐specific exosomes reveals joint pathways of colorectal cancer in both clinical samples and cell culture. Cancer Letters, 469, 186–194. 31669517 10.1016/j.canlet.2019.10.038

[jev212027-bib-0022] Fabbri, M. , Paone, A. , Calore, F. , Galli, R. , Gaudio, E. , Santhanam, R. , … Croce, C. M. (2012). MicroRNAs bind to Toll‐like receptors to induce prometastatic inflammatory response. PNAS, 109(31), E2110–E2116. 22753494 10.1073/pnas.1209414109 PMC3412003

[jev212027-bib-0023] Fernández‐Arjona, M. D. M. , Grondona, J. M. , Fernández‐Llebrez, P. , & López‐Ávalos, M. D. (2019). Microglial Morphometric Parameters Correlate With the Expression Level of IL‐1beta, and Allow Identifying Different Activated Morphotypes. Front Cell Neurosci, 13, 472. 31708746 10.3389/fncel.2019.00472 PMC6824358

[jev212027-bib-0024] Fernández‐Arjona, M. D. M. , Grondona, J. M. , Granados‐Durán, P. , Fernández‐Llebrez, P. , & López‐Ávalos, M. D. (2017). Microglia morphological categorization in a rat model of neuroinflammation by hierarchical cluster and principal components analysis. Front Cell Neurosci, 11, 235. 28848398 10.3389/fncel.2017.00235 PMC5550745

[jev212027-bib-0025] Filipowicz, W. , Bhattacharyya, S. N. , & Sonenberg, N. (2008). Mechanisms of post‐transcriptional regulation by microRNAs: Are the answers in sight? Nature Reviews Genetics, 9(2), 102–114. 10.1038/nrg2290 18197166

[jev212027-bib-0026] Fitting, S. , Knapp, P. E. , Zou, S. , Marks, W. D. , Bowers, M. S. , Akbarali, H. I. , & Hauser, K. F. (2014). Interactive HIV‐1 Tat and morphine‐induced synaptodendritic injury is triggered through focal disruptions in Na(+) influx, mitochondrial instability, and Ca(2)(+) overload. Journal of Neuroscience, 34(38), 12850–12864. 25232120 10.1523/JNEUROSCI.5351-13.2014 PMC4166164

[jev212027-bib-0027] Gambuzza, M. , Sofo, V. , Salmeri, F. , Soraci, L. , Marino, S. , & Bramanti, P. (2014). Toll‐like receptors in Alzheimer's disease: A therapeutic perspective. CNS Neurol Disord Drug Targets, 13(9), 1542–1558. 25106635 10.2174/1871527313666140806124850

[jev212027-bib-0028] Gámez‐Valero, A. , Beyer, K. , & Borràs, F. E. (2019). Extracellular vesicles, new actors in the search for biomarkers of dementias. Neurobiology of Aging, 74, 15–20. 30396120 10.1016/j.neurobiolaging.2018.10.006

[jev212027-bib-0029] Gao, T. , Shu, J. , & Cui, J. (2018). A systematic approach to RNA‐associated motif discovery. Bmc Genomics [Electronic Resource], 19(1), 146. 10.1186/s12864-018-4528-x PMC5813387 29444662

[jev212027-bib-0030] Gayen, M. , Bhomia, M. , Balakathiresan, N. , & Knollmann‐Ritschel, B. (2020). Exosomal MicroRNAs Released by Activated Astrocytes as Potential Neuroinflammatory Biomarkers. International Journal of Molecular Sciences, 2312, 21(7) 10.3390/ijms21072312 PMC7177648 32230793

[jev212027-bib-0031] Guo, M.‐L. , Periyasamy, P. , Liao, Ke , Kook, Y. H. , Niu, F. , Callen, S. E. , & Buch, S. (2016). Cocaine‐mediated downregulation of microglial miR‐124 expression involves promoter DNA methylation. Epigenetics, 11(11), 819–830. 27786595 10.1080/15592294.2016.1232233 PMC5221603

[jev212027-bib-0032] Gupta, A. , & Pulliam, L. (2014). Exosomes as mediators of neuroinflammation. J Neuroinflammation, 11, 68. 24694258 10.1186/1742-2094-11-68 PMC3994210

[jev212027-bib-0033] Gurwell, J. A. , Nath, A. , Sun, Q. , Zhang, J. , Martin, K. M. , Chen, Y. , & Hauser, K. F. (2001). Synergistic neurotoxicity of opioids and human immunodeficiency virus‐1 Tat protein in striatal neurons in vitro. Neuroscience, 102(3), 555–563. 11226693 10.1016/s0306-4522(00)00461-9 PMC4300203

[jev212027-bib-0034] Hahn, Y. K. , Paris, J. J. , Lichtman, A. H. , Hauser, K. F. , Sim‐Selley, L. J. , Selley, D. E. , & Knapp, P. E. (2016). Central HIV‐1 Tat exposure elevates anxiety and fear conditioned responses of male mice concurrent with altered mu‐opioid receptor‐mediated G‐protein activation and beta‐arrestin 2 activity in the forebrain. Neurobiology of Disease, 92(Pt B), 124–136. 26845176 10.1016/j.nbd.2016.01.014 PMC4907901

[jev212027-bib-0035] Haroutounian, S. (2018). Postoperative opioids, endocrine changes, and immunosuppression. Pain Reports, 3(2), e640. 29756086 10.1097/PR9.0000000000000640 PMC5902248

[jev212027-bib-0036] Harrison, E. B. , Hochfelder, C. G. , Lamberty, B. G. , Meays, B. M. , Morsey, B. M. , Kelso, M. L. , … Yelamanchili, S. V. (2016). Traumatic brain injury increases levels of miR‐21 in extracellular vesicles: Implications for neuroinflammation. FEBS Open Bio, 6(8), 835–846. 10.1002/2211-5463.12092 PMC4971839 27516962

[jev212027-bib-0037] Heil, F. (2004). Species‐specific recognition of single‐stranded RNA via toll‐like receptor 7 and 8. Science, 303(5663), 1526–1529. 14976262 10.1126/science.1093620

[jev212027-bib-0038] Herkenham, M. , & Pert, Cb (1982). Light microscopic localization of brain opiate receptors: A general autoradiographic method which preserves tissue quality. Journal of Neuroscience, 2(8), 1129–1149. 6286904 10.1523/JNEUROSCI.02-08-01129.1982 PMC6564275

[jev212027-bib-0039] Hernández, J. C. , Stevenson, M. , Latz, E. , & Urcuqui‐Inchima, S. (2012). HIV type 1 infection up‐regulates TLR2 and TLR4 expression and function in vivo and in vitro. Aids Research and Human Retroviruses, 28(10), 1313–1328. 22280204 10.1089/aid.2011.0297 PMC3482876

[jev212027-bib-0040] Hu, G. , Liao, K. , Yang, L. , Pendyala, G. , Kook, Y. , Fox, H. S. , & Buch, S. (2017). Tat‐Mediated Induction of miRs‐34a & ‐138 Promotes Astrocytic Activation via Downregulation of SIRT1: Implications for Aging in HAND. Journal of neuroimmune pharmacology : the official journal of the Society on NeuroImmune Pharmacology, 12(3), 420–432. 28236278 10.1007/s11481-017-9730-0 PMC5546000

[jev212027-bib-0041] Hu, G. , Liao, Ke , Niu, F. , Yang, Lu , Dallon, B. W. , Callen, S. , … Buch, S. (2018). Astrocyte EV‐induced lincRNA‐Cox2 regulates microglial phagocytosis: implications for morphine‐mediated neurodegeneration. Molecular Therapy. Nucleic Acids, 13, 450–463. 30388619 10.1016/j.omtn.2018.09.019 PMC6202788

[jev212027-bib-0042] Hu, G. , Niu, F. , Liao, K. , Periyasamy, P. , Sil, S. , Liu, J. , … Buch, S. (2019). HIV‐1 Tat‐induced astrocytic extracellular vesicle miR‐7 impairs synaptic architecture. Journal of Neuroimmune Pharmacology : The official journal of the Society on NeuroImmune Pharmacology, 15, 538–553. 31401755 10.1007/s11481-019-09869-8 PMC7008083

[jev212027-bib-0043] Hu, G. , Niu, F. , Liao, Ke , Periyasamy, P. , Sil, S. , Liu, J. , … Buch, S. (2020). HIV‐1 Tat‐induced astrocytic extracellular vesicle miR‐7 impairs synaptic architecture. Journal of Neuroimmune Pharmacology : The official journal of the Society on NeuroImmune Pharmacology, 15(3), 538–553. 31401755 10.1007/s11481-019-09869-8 PMC7008083

[jev212027-bib-0044] Hu, G. , Yao, H. , Chaudhuri, A. D. , Duan, M. , Yelamanchili, S. V. , Wen, H. , … Buch, S. (2012). Exosome‐mediated shuttling of microRNA‐29 regulates HIV Tat and morphine‐mediated neuronal dysfunction. Cell Death and Disease, 3, e381. 22932723 10.1038/cddis.2012.114 PMC3434655

[jev212027-bib-0045] Hutchinson, M. R. , Lewis, S. S. , Coats, B. D. , Skyba, D. A. , Crysdale, N. Y. , Berkelhammer, D. L. , … Johnson, K. W. (2009). Reduction of opioid withdrawal and potentiation of acute opioid analgesia by systemic AV411 (ibudilast). Brain, Behavior, and Immunity, 23(2), 240–250. 10.1016/j.bbi.2008.09.012 PMC2662518 18938237

[jev212027-bib-0046] Ibáñez, F. , Montesinos, J. , Ureña‐Peralta, J. R. , Guerri, C. , & Pascual, M. (2019). TLR4 participates in the transmission of ethanol‐induced neuroinflammation via astrocyte‐derived extracellular vesicles. J Neuroinflammation, 16(1), 136. 31272469 10.1186/s12974-019-1529-x PMC6610989

[jev212027-bib-0047] Joyce, D. P. , Kerin, M. J. , & Dwyer, R. M. (2016). Exosome‐encapsulated microRNAs as circulating biomarkers for breast cancer. International Journal of Cancer, 139(7), 1443–1448. 27170104 10.1002/ijc.30179

[jev212027-bib-0048] Kim, Ji Y. , Ahn, H. J. , Kim, J. K. , Kim, J. , Lee, S. H. , & Chae, H. B. (2016). Morphine suppresses lung cancer cell proliferation through the interaction with opioid growth factor receptor: An in vitro and human lung tissue study. Anesthesia and Analgesia, 123(6), 1429–1436. 27167686 10.1213/ANE.0000000000001293

[jev212027-bib-0049] Lee, K. , Vuong, H. E. , Nusbaum, D. J. , Hsiao, E. Y. , Evans, C. J. , & Taylor, A. M. W. (2018). The gut microbiota mediates reward and sensory responses associated with regimen‐selective morphine dependence. Neuropsychopharmacology, 43(13), 2606–2614. 30258112 10.1038/s41386-018-0211-9 PMC6224506

[jev212027-bib-0050] Lehmann, S. M. , Krüger, C. , Park, B. , Derkow, K. , Rosenberger, K. , Baumgart, J. , … Lehnardt, S. (2012). An unconventional role for miRNA: Let‐7 activates Toll‐like receptor 7 and causes neurodegeneration. Nature Neuroscience, 15(6), 827–835. 22610069 10.1038/nn.3113

[jev212027-bib-0051] Lewis, J. M. , Vyas, A. D. , Qiu, Y. , Messer, K. S. , White, R. , & Heller, M. J. (2018). Integrated analysis of exosomal protein biomarkers on alternating current electrokinetic chips enables rapid detection of pancreatic cancer in patient blood. ACS Nano, 12(4), 3311–3320. 29570265 10.1021/acsnano.7b08199

[jev212027-bib-0052] Li, Y. , Merrill, J. D. , Mooney, K. , Song, Li , Wang, Xu , Guo, C.‐J. , … Ho, W.‐Z. (2003). Morphine enhances HIV infection of neonatal macrophages. Pediatric Research, 54(2), 282–288. 12736382 10.1203/01.PDR.0000074973.83826.4C PMC4035124

[jev212027-bib-0053] Liao, Ke , Guo, M. , Niu, F. , Yang, Lu , Callen, S. E. , & Buch, S. (2016). Cocaine‐mediated induction of microglial activation involves the ER stress‐TLR2 axis. Journal of Neuroinflammation, 13, 33. 26860188 10.1186/s12974-016-0501-2 PMC4748483

[jev212027-bib-0054] Liu, C. , Xu, X. , Li, Bo , Situ, Bo , Pan, W. , Hu, Yu , … Zheng, L. (2018). Single‐exosome‐counting immunoassays for cancer diagnostics. Nano Letters, 18(7), 4226–4232. 29888919 10.1021/acs.nanolett.8b01184

[jev212027-bib-0055] Liu, H. , Zhou, R. H. , Liu, Y. , Guo, L. , Wang, X. , Hu, W. H. , & Ho, W. Z. (2020). HIV infection suppresses TLR3 activation‐mediated antiviral immunity in microglia and macrophages. Immunology, 160, 269–279. 32053234 10.1111/imm.13181 PMC7341545

[jev212027-bib-0056] Liu, L. , Zuo, L. , Yang, J. , Xin, S. , Zhang, J. , Zhou, J. , … Lu, J. (2019). Exosomal cyclophilin A as a novel noninvasive biomarker for Epstein‐Barr virus associated nasopharyngeal carcinoma. Cancer medicine, 8(6), 3142–3151. 31063269 10.1002/cam4.2185 PMC6558463

[jev212027-bib-0057] Matzeu, A. , Zamora‐Martinez, E. R. , & Martin‐Fardon, R. (2014). The paraventricular nucleus of the thalamus is recruited by both natural rewards and drugs of abuse: Recent evidence of a pivotal role for orexin/hypocretin signaling in this thalamic nucleus in drug‐seeking behavior. Frontiers in Behavioral Neuroscience, 8, 117. 24765071 10.3389/fnbeh.2014.00117 PMC3982054

[jev212027-bib-0058] Mcnitt, J. I. , & Lukefahr, S. D. (1990). Effects of breed, parity, day of lactation and number of kits on milk production of rabbits. Journal of Animal Science, 68(6), 1505–1512. 2384351 10.2527/1990.6861505x

[jev212027-bib-0059] Men, Y. , Yelick, J. , Jin, S. , Tian, Y. , Chiang, M. S. R. , Higashimori, H. , … Yang, Y. (2019). Exosome reporter mice reveal the involvement of exosomes in mediating neuron to astroglia communication in the CNS. NATURE communications, 10(1), 4136. 10.1038/s41467-019-11534-w PMC6742670 31515491

[jev212027-bib-0060] Miller, S. M. , Cybulski, V. , Whitacre, M. , Bess, L. S. , Livesay, M. T. , Walsh, L. , … Evans, J. T. (2020). Novel lipidated imidazoquinoline TLR7/8 adjuvants elicit influenza‐specific Th1 immune responses and protect against heterologous H3N2 influenza challenge in mice. Frontiers in immunology, 11, 406. 32210973 10.3389/fimmu.2020.00406 PMC7075946

[jev212027-bib-0061] Ñahui Palomino, R. A. , Vanpouille, C. , Laghi, L. , Parolin, C. , Melikov, K. , Backlund, P. , … Margolis, L. (2019). Extracellular vesicles from symbiotic vaginal lactobacilli inhibit HIV‐1 infection of human tissues. Nature communications, 10(1), 5656. 10.1038/s41467-019-13468-9 PMC6906448 31827089

[jev212027-bib-0062] NIH (2019) Overdose Death Rates. (NIH).

[jev212027-bib-0063] Nogueras‐Ortiz, C. J. , Mahairaki, V. , Delgado‐Peraza, F. , Das, D. , Avgerinos, K. , Eren, E. , … Kapogiannis, D. (2020). Astrocyte‐ and neuron‐derived extracellular vesicles from Alzheimer's disease patients effect complement‐mediated neurotoxicity. Cells, 9(7), 1618 10.3390/cells9071618 PMC7407141 32635578

[jev212027-bib-0064] Paolicelli, R. C. , Bergamini, G. , & Rajendran, L. (2019). Cell‐to‐cell communication by extracellular vesicles: focus on microglia. Neuroscience, 405, 148–157. 29660443 10.1016/j.neuroscience.2018.04.003

[jev212027-bib-0065] Periyasamy, P. , Liao, Ke , Kook, Y. H. , Niu, F. , Callen, S. E. , Guo, M. ‐. L. , & Buch, S. (2018). Cocaine‐mediated downregulation of miR‐124 activates microglia by targeting KLF4 and TLR4 signaling. Molecular Neurobiology, 55(4), 3196–3210. 28478506 10.1007/s12035-017-0584-5 PMC5673594

[jev212027-bib-0066] Reddy, P. V. , Pilakka‐Kanthikeel, S. , Saxena, S. K. , Saiyed, Z. , & Nair, M. P. (2012). Interactive effects of morphine on HIV infection: Role in HIV‐associated neurocognitive disorder. AIDS Research and Treatment, 2012, 1. 10.1155/2012/953678 PMC3362817 22666564

[jev212027-bib-0067] Rodrigues, M. , Fan, J. , Lyon, C. , Wan, M. , & Hu, Ye (2018). Role of extracellular vesicles in viral and bacterial infections: Pathogenesis, diagnostics, and therapeutics. Theranostics, 8(10), 2709–2721. 29774070 10.7150/thno.20576 PMC5957004

[jev212027-bib-0068] Roy, J. , Saucier, D. , O'connell, C. , & Morin, P. J.r (2019). Extracellular vesicles and their diagnostic potential in amyotrophic lateral sclerosis. Clinica Chimica Acta, 497, 27–34. 10.1016/j.cca.2019.07.012 31301281

[jev212027-bib-0069] Russell, A. E. , Jun, S. , Sarkar, S. , Geldenhuys, W. J. , Lewis, S. E. , Rellick, S. L. , & Simpkins, J. W. (2019). Extracellular vesicles secreted in response to cytokine exposure increase mitochondrial oxygen consumption in recipient cells. Front Cell Neurosci, 13, 51. 30837842 10.3389/fncel.2019.00051 PMC6383587

[jev212027-bib-0070] Sampey, G. C. , Saifuddin, M. , Schwab, A. , Barclay, R. , Punya, S. , Chung, M. ‐. C. , … Kashanchi, F. (2016). Exosomes from HIV‐1‐infected cells stimulate production of pro‐inflammatory cytokines through trans‐activating response (TAR) RNA. Journal of Biological Chemistry, 291(3), 1251–1266. 10.1074/jbc.M115.662171 PMC4714213 26553869

[jev212027-bib-0071] Sardar Sinha, M. , Ansell‐Schultz, A. , Civitelli, L. , Hildesjö, C. , Larsson, M. , Lannfelt, L. , … Hallbeck, M. (2018). Alzheimer's disease pathology propagation by exosomes containing toxic amyloid‐beta oligomers. Acta Neuropathologica, 136(1), 41–56. 29934873 10.1007/s00401-018-1868-1 PMC6015111

[jev212027-bib-0072] Selmaj, I. , Mycko, M. P. , Raine, C. S. , & Selmaj, K. W. (2017). The role of exosomes in CNS inflammation and their involvement in multiple sclerosis. Journal of Neuroimmunology, 306, 1–10. 28385180 10.1016/j.jneuroim.2017.02.002

[jev212027-bib-0073] Shen, J. , Huang, C. ‐. K. , Yu, H. , Shen, Bo , Zhang, Y. , Liang, Y. , … Cai, X. (2017). The role of exosomes in hepatitis, liver cirrhosis and hepatocellular carcinoma. Journal of Cellular and Molecular Medicine, 21(5), 986–992. 28224705 10.1111/jcmm.12950 PMC5387156

[jev212027-bib-0074] Shi, M. , Liu, C. , Cook, T. J. , Bullock, K. M. , Zhao, Y. , Ginghina, C. , … Zhang, J. (2014). Plasma exosomal alpha‐synuclein is likely CNS‐derived and increased in Parkinson's disease. Acta Neuropathologica, 128(5), 639–650. 24997849 10.1007/s00401-014-1314-y PMC4201967

[jev212027-bib-0075] Sim, L. J. , Selley, D. E. , & Childers, S. R. (1995). In vitro autoradiography of receptor‐activated G proteins in rat brain by agonist‐stimulated guanylyl 5'‐[gamma‐[35S]thio]‐triphosphate binding. PNAS, 92(16), 7242–7246. 7638174 10.1073/pnas.92.16.7242 PMC41315

[jev212027-bib-0076] Sullivan, M. D. (2018). Depression Effects on Long‐term Prescription Opioid Use, Abuse, and Addiction. Clinical Journal of Pain, 34(9), 878–884. 10.1097/AJP.0000000000000603 29505419

[jev212027-bib-0077] Sun, Z. , Shi, Ke , Yang, S. , Liu, J. , Zhou, Q. , Wang, G. , … Yuan, W. (2018). Effect of exosomal miRNA on cancer biology and clinical applications. Molecular Cancer [Electronic Resource], 17(1), 147. 10.1186/s12943-018-0897-7 PMC6182840 30309355

[jev212027-bib-0078] Terrett, G. , Mclennan, S. N. , Henry, J. D. , Biernacki, K. , Mercuri, K. , Curran, H. V. , & Rendell, P. G. (2014). Prospective memory impairment in long‐term opiate users. Psychopharmacology, 231(13), 2623–2632. 24448901 10.1007/s00213-014-3432-6

[jev212027-bib-0079] Tkach, M. , & Théry, C. (2016). Communication by extracellular vesicles: Where we are and where we need to go. Cell, 164(6), 1226–1232. 26967288 10.1016/j.cell.2016.01.043

[jev212027-bib-0080] Vasko, J. S. , Henney, R. P. , Oldham, H. N. , Brawley, R. K. , & Morrow, A. G. (1966). Mechanisms of action of morphine in the treatment of experimental pulmonary edema. American Journal of Cardiology, 18(6), 876–883. 10.1016/0002-9149(66)90433-4 5923998

[jev212027-bib-0081] Venturini, A. , Passalacqua, M. , Pelassa, S. , Pastorino, F. , Tedesco, M. , Cortese, K. , … Cervetto, C. (2019). Exosomes From Astrocyte Processes: Signaling to Neurons. Front Pharmacol, 10, 1452. 31849688 10.3389/fphar.2019.01452 PMC6901013

[jev212027-bib-0082] Vu, L. T. , Peng, B. , Zhang, D. X. , Ma, V. , Mathey‐Andrews, C. A. , Lam, C. K. , … Le, M. T.n (2019). Tumor‐secreted extracellular vesicles promote the activation of cancer‐associated fibroblasts via the transfer of microRNA‐125b. Journal of extracellular vesicles, 8(1), 1599680. 31044053 10.1080/20013078.2019.1599680 PMC6484490

[jev212027-bib-0083] Wang, F. , Meng, J. , Zhang, Li , Johnson, T. , Chen, C. , & Roy, S. (2018). Morphine induces changes in the gut microbiome and metabolome in a morphine dependence model. Scientific Reports, 8(1), 3596. 29483538 10.1038/s41598-018-21915-8 PMC5827657

[jev212027-bib-0084] Wang, J. B. , Imai, Y. , Eppler, C. M. , Gregor, P. , Spivak, C. E. , & Uhl, G. R. (1993). mu opiate receptor: CDNA cloning and expression. PNAS, 90(21), 10230–10234. 8234282 10.1073/pnas.90.21.10230 PMC47748

[jev212027-bib-0085] Wang, W. Y. , Tan, M. S. , Yu, J. T. , & Tan, L. (2015). Role of pro‐inflammatory cytokines released from microglia in Alzheimer's disease. Annals of translational medicine, 3(10), 136. 26207229 10.3978/j.issn.2305-5839.2015.03.49 PMC4486922

[jev212027-bib-0086] Willis, C. M. , Nicaise, A. M. , Bongarzone, E. R. , Givogri, M. , Reiter, C. R. , Heintz, O. , … Crocker, S. J. (2020). Astrocyte support for oligodendrocyte differentiation can be conveyed via extracellular vesicles but diminishes with age. Scientific Reports, 10(1), 828. 31964978 10.1038/s41598-020-57663-x PMC6972737

[jev212027-bib-0087] Wu, W. , Dietze, K. K. , Gibbert, K. , Lang, K. S. , Trilling, M. , Yan, H. , … Liu, J. (2015). TLR ligand induced IL‐6 counter‐regulates the anti‐viral CD8(+) T cell response during an acute retrovirus infection. Scientific Reports, 5, 10501. 25994622 10.1038/srep10501 PMC4440206

[jev212027-bib-0088] Xia, X. , Wang, Yi , Huang, Y. , Zhang, H. , Lu, H. , & Zheng, J. C. (2019). Exosomal miRNAs in central nervous system diseases: Biomarkers, pathological mediators, protective factors and therapeutic agents. Progress in Neurobiology, 183, 101694. 31542363 10.1016/j.pneurobio.2019.101694 PMC7323939

[jev212027-bib-0089] Xiang, B. , Zhong, P. , Fang, L. , Wu, X. , Song, Y. , & Yuan, H. (2019). miR‐183 inhibits microglia activation and expression of inflammatory factors in rats with cerebral ischemia reperfusion via NF‐kappaB signaling pathway. Exp Ther Med, 18(4), 2540–2546. 31572505 10.3892/etm.2019.7827 PMC6755485

[jev212027-bib-0090] Yang, Lu , Niu, F. , Yao, H. , Liao, Ke , Chen, X. , Kook, Y. , … Buch, S. (2018). Exosomal miR‐9 Released from HIV Tat Stimulated Astrocytes Mediates Microglial Migration. J Neuroimmune Pharmacol, 13(3), 330–344. 29497921 10.1007/s11481-018-9779-4 PMC6082702

[jev212027-bib-0091] Yelamanchili, S. V. , Lamberty, B. G. , Rennard, D. A. , Morsey, B. M. , Hochfelder, C. G. , Meays, B. M. , … Fox, H. S. (2015). MiR‐21 in Extracellular Vesicles Leads to Neurotoxicity via TLR7 Signaling in SIV Neurological Disease. Plos Pathogens, 11(7), e1005032. 26154133 10.1371/journal.ppat.1005032 PMC4496044

[jev212027-bib-0092] Young, K. , & Morrison, H. (2018). Quantifying microglia morphology from photomicrographs of immunohistochemistry prepared tissue using imageJ. Journal of Visualized Experiments: JoVE, (136), e57648 10.3791/57648 PMC6103256 29939190

[jev212027-bib-0093] Zhang, L. , Zhang, J. , & You, Z. (2018). Switching of the Microglial Activation Phenotype Is a Possible Treatment for Depression Disorder. Front Cell Neurosci, 12, 306. 30459555 10.3389/fncel.2018.00306 PMC6232769

[jev212027-bib-0094] Zhang, Li , Meng, J. , Ban, Y. , Jalodia, R. , Chupikova, I. , Fernandez, I. , … Roy, S. (2019). Morphine tolerance is attenuated in germfree mice and reversed by probiotics, implicating the role of gut microbiome. PNAS, 116(27), 13523–13532. 31209039 10.1073/pnas.1901182116 PMC6613141

[jev212027-bib-0095] Zhang, Z. , Ohto, U. , Shibata, T. , Krayukhina, E. , Taoka, M. , Yamauchi, Y. , … Shimizu, T. (2016). Structural analysis reveals that toll‐like receptor 7 is a dual receptor for guanosine and single‐stranded RNA. Immunity, 45(4), 737–748. 27742543 10.1016/j.immuni.2016.09.011

