# Supplementary figures and images for: Morphine‐mediated release of miR‐138 in astrocyte‐derived extracellular vesicles promotes microglial activation
Source: J Extracell Vesicles. 2020 Nov 19;10(1):e12027. doi: 10.1002/jev2.12027 (PMC7710131; doi:10.1002/jev2.12027)

**A.**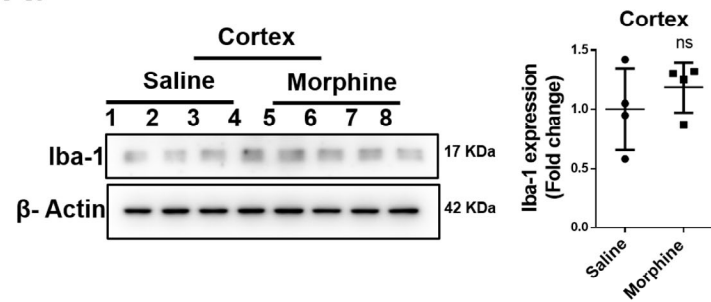**B.**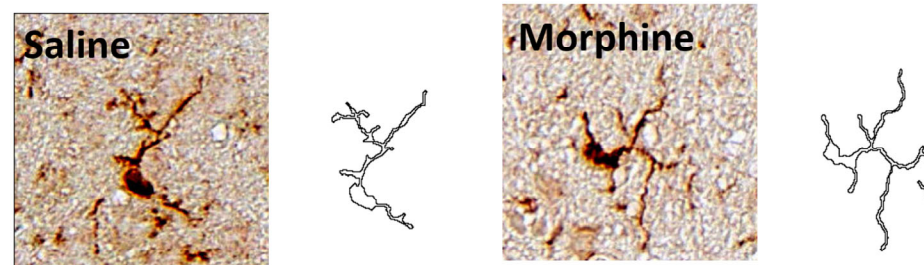**C.**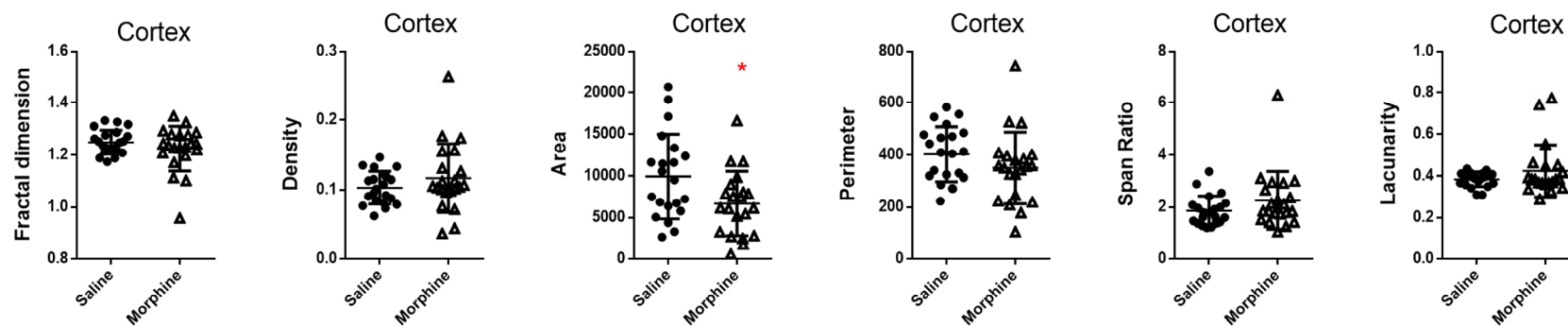**D.**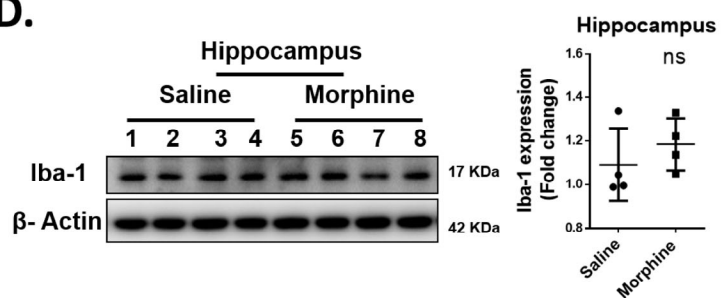**E.**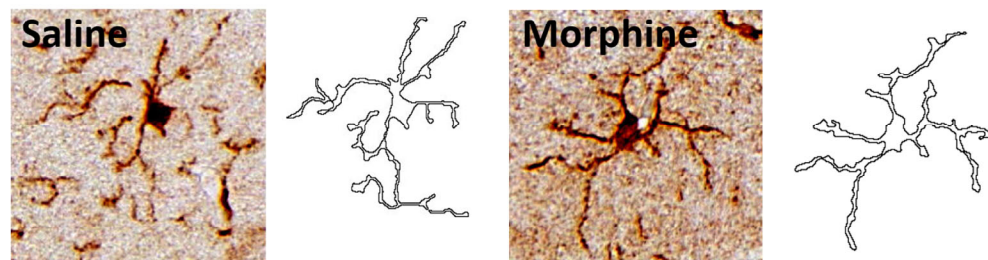**F.**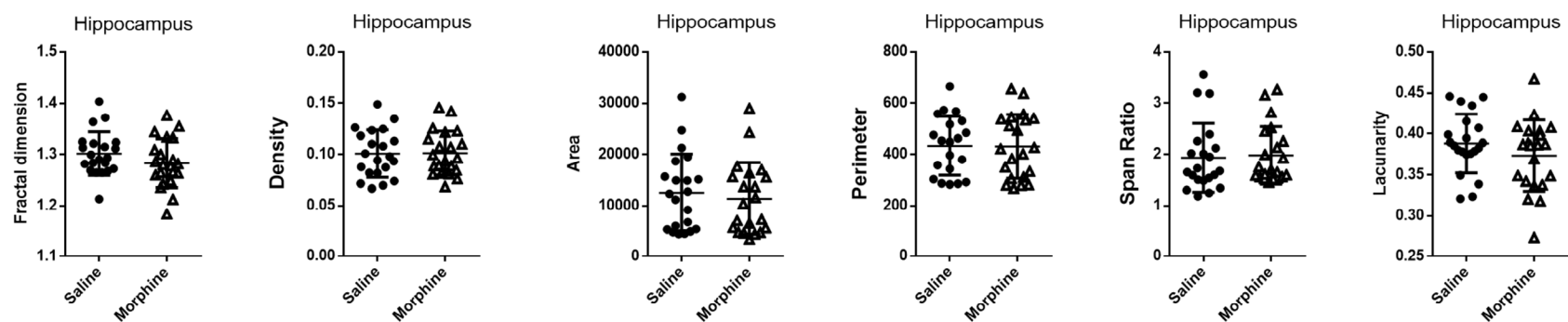

Supplement: Supplementary file 1 — SFigure. 1 Morphine‐induced microglial activation. (A) Representative western blot and quantification of Iba1 in the lysates of cortices of mice administrated saline or morphine (n = 4/group). (B) Representative images of Iba‐1+ cells and pairwise outline shapes were used for morphological parameters measures in the cortices of mice administrated saline or morphine. (C) Quantification of morphological parameters fractal dimension, density, area, perimeter, span ratio, and lacunarity in cortices of mice administrated saline or morphine (n = 5 or 6/animal). (D) Representative western blot and quantification of Iba1 in the hippocampus lysates of mice administrated saline or morphine. (E) Representative images of Iba‐1+ cells and pairwise outline shapes were used for morphological parameters measures in the hippocampi of mice administrated saline or morphine. (F) Quantification of morphological parameters: fractal dimension, density, area, perimeter, span ratio and lacunarity in the hippocampi of mice administrated saline or morphine (n = 5 or 6/animal). All data are presented as mean ± SD or SEM of four individual experiments. *, p < 0.05; **, p < 0.01; ***, p < 0.001 versus saline group using Student's t test. [file JEV2-10-e12027-s001.pdf]

A.

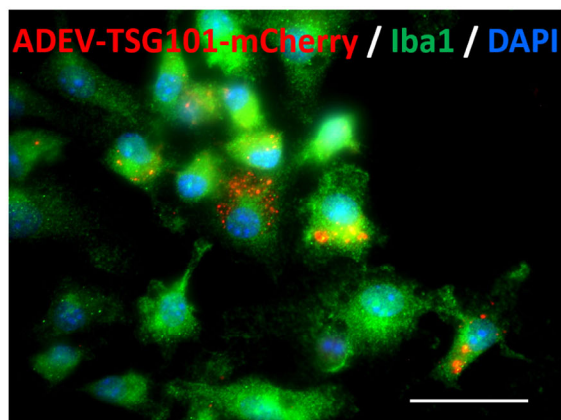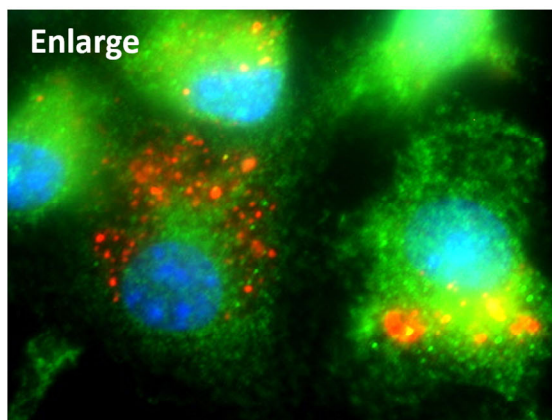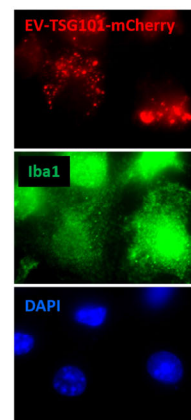

B.

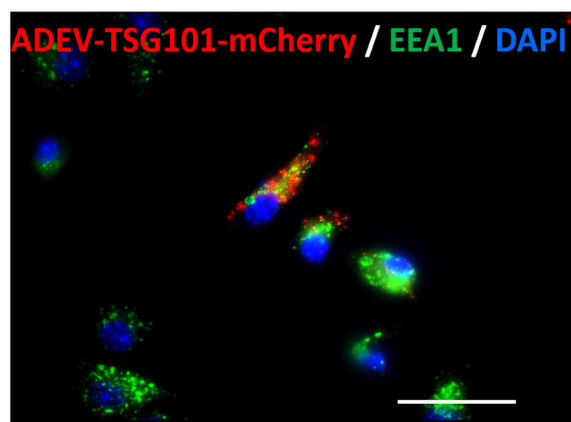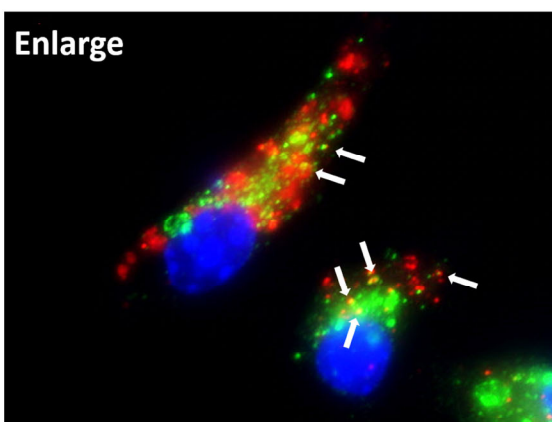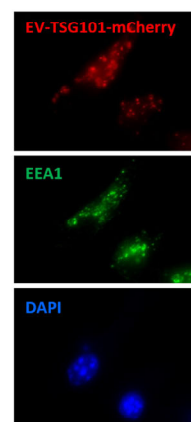

C. ADEV + (Cy5-miR138+RNaseA)

(ADEV-Cy5-miR138) + RNaseA

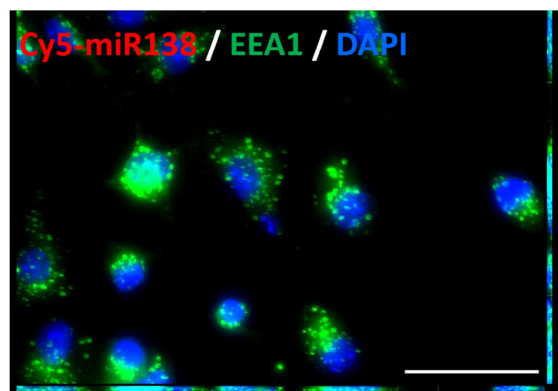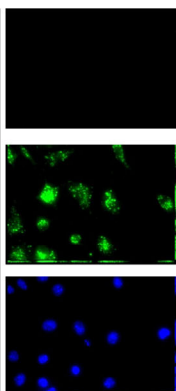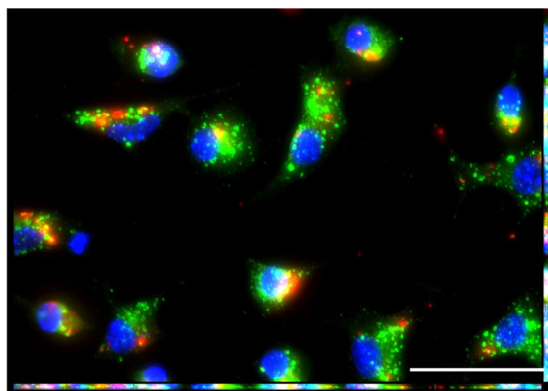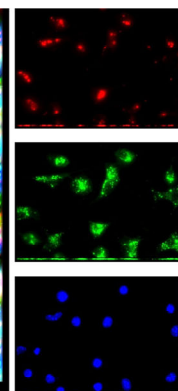

Supplement: Supplementary file 3 — SFigure 3. EVs are internalized by the microglia and reach the endosomes. (A & B) Representative fluorescence images of mouse primary microglial cells incubated with EVs purified from pEF6.mCherry‐TSG101‐transfected mouse primary astrocytes for 30 min, followed by immunostaining of microglial marker Iba‐1 (A) and the early endosome marker (EEA1) (B). Iba1 (Green), EEA1(Green), EV‐TSG101‐mCherry (Red). Bars, 30 μm (n = 3). (C) Representative fluorescence images of mouse primary microglial cells incubated with ADEV‐Cy5‐miR138+RNaseA (Cy5‐miR138 was first loaded into the ADEV followed by the incubation with RNase) or ADEV+RNaseA‐Cy5‐miR138 (Cy5‐miR138 was first incubated with RNase followed by loaded into ADEVs) followed by immunostaining MPMs for the endosomal marker EEA1. Bars, 30 μm (n = 3). [file JEV2-10-e12027-s003.pdf]

A.

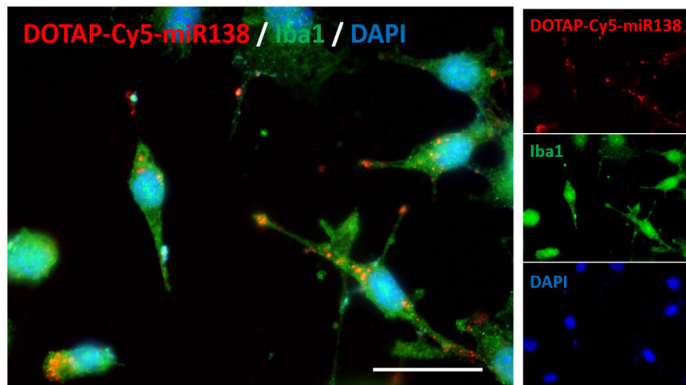

B.

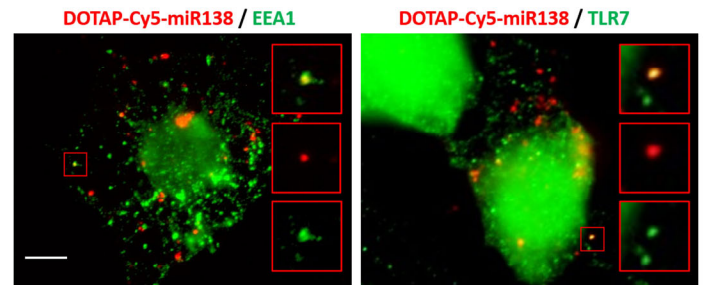

C.

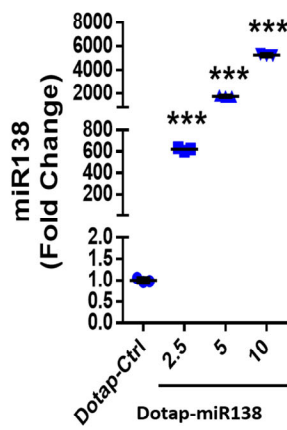

D.

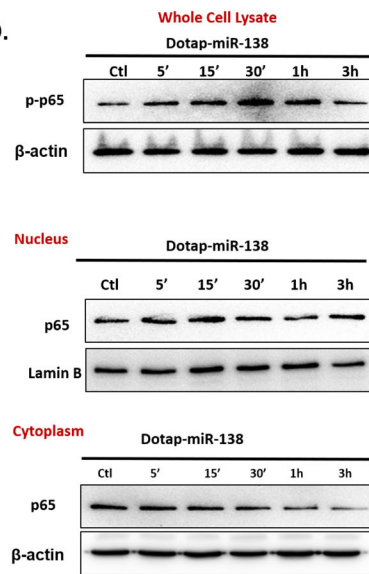

E.

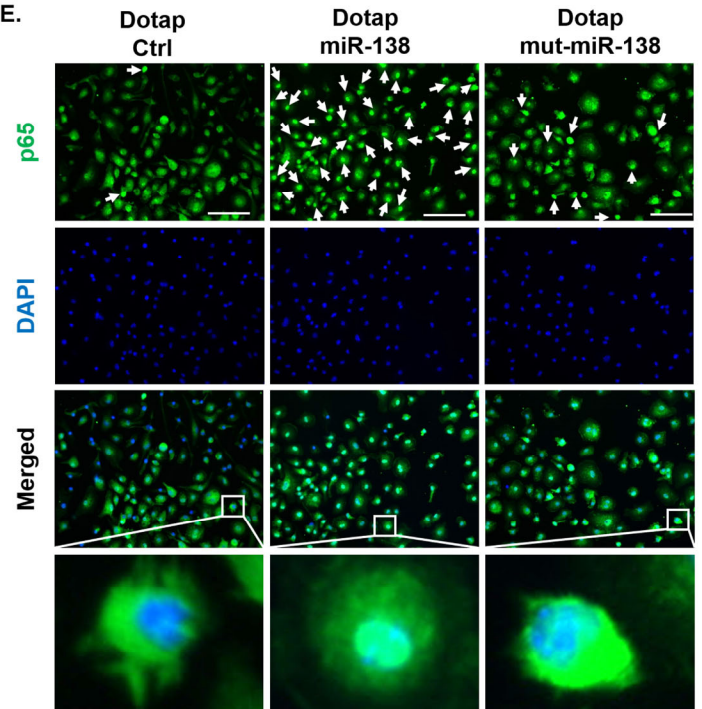

F.

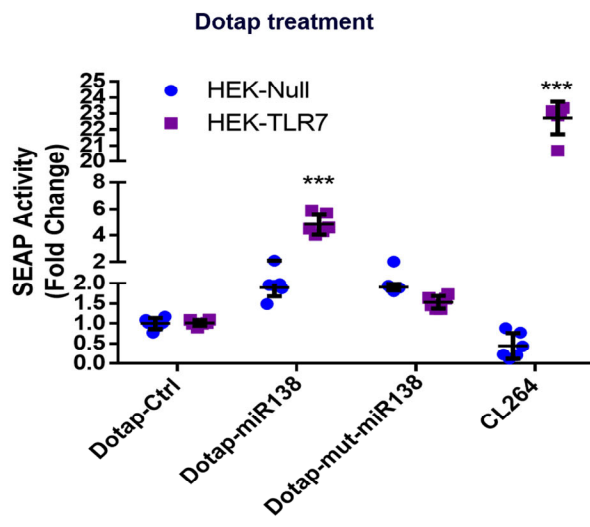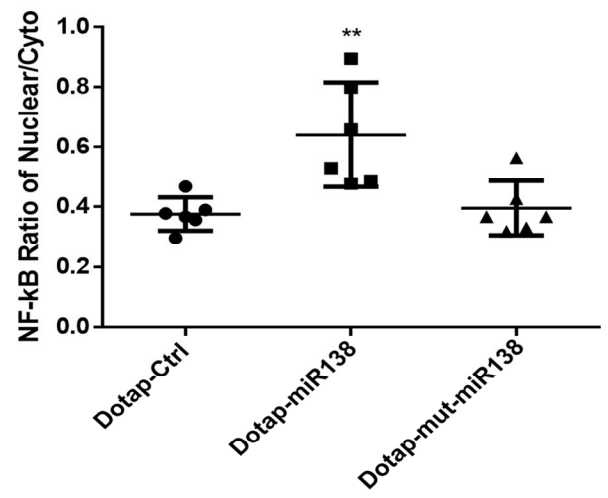

Supplement: Supplementary file 4 — SFigure 4. Dotap‐miR138 mediated NF‐kb nuclear translocation in BV2 cells. (A) Representative fluorescence images of BV2 cells transfected with DOTAP‐Cy5‐miR138 for 30 min, followed by the immunostaining of (A) microglial marker Iba1 (Green), Bars, 20 μm, (B) early endosome marker EEA1 (Green) and TLR7 (Green). Bars, 5 μm (n = 3). (C) Real‐time PCR analysis of miR138 mRNA expression in BV2 cells transfected with various concentration of DOTAP‐miR138 (2.5, 5, 10 pmol / well) (n = 3). (D) Representative western blot of p‐NF‐κB p65 in the lysates or NF‐κB p65 in the nuclear and cytoplasmic lysates isolated from BV2 cells transfected with DOTAP‐miR‐138 for various time points (5 min to 3 h) (n = 3). (E) BV2 cells were transfected with DOTAP‐miR‐138 or DOTAP‐mut‐miR‐138, followed by immunostaining with antibodies specific for NF‐κB p65. Bars, 30 μm. White arrows, NF‐κB nuclear translocated cells (n = 3, two images were analyzed each experiment). (F) SEAP activity in HEK‐Null / HEK‐TLR7 cells exposed to DOTAP‐miR‐138 or DOTAP‐mut‐miR‐138 or CL264 (n = 3, two wells were tested each experiment). All data are presented as mean ± SD or SEM of three individual experiments. **, p < 0.01; ***, p < 0.001 versus control group. [file JEV2-10-e12027-s004.pdf]

**A.****Cytoplasm**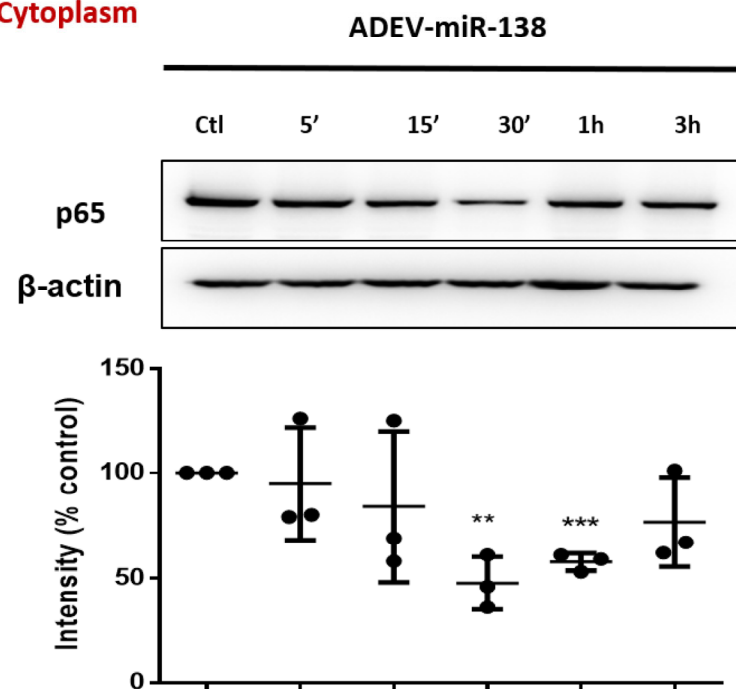**B.****Nucleus**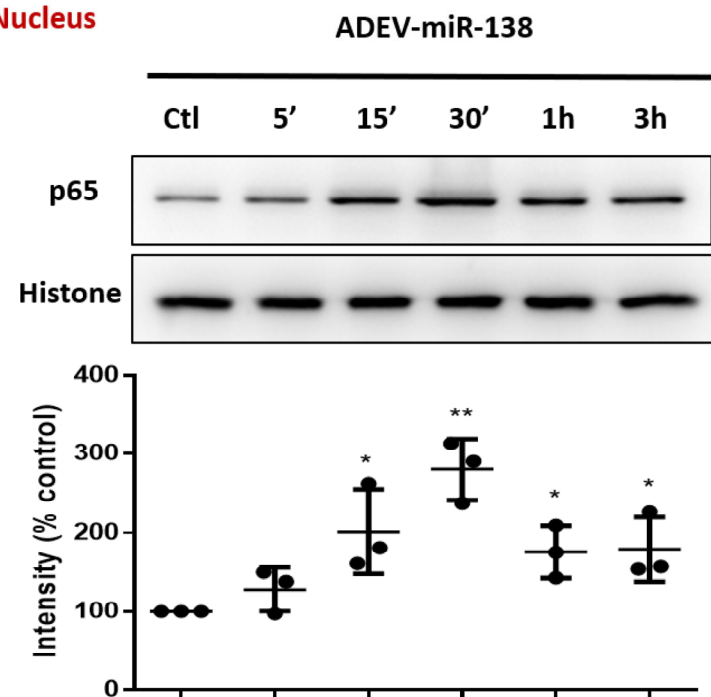

Supplement: Supplementary file 5 — SFigure 5. ADEV‐miR138 mediated NF‐kb (p65) nuclear translocation in MPMs. (A, B) Representative western blot of NF‐κB p65 in the cytoplasmic (A) and nuclear lysates (B) isolated from MPMs exposed to ADEV‐miR‐138 for various time points (5 min to 3 h). All data are presented as mean ± SD or SEM of three individual experiments. *, p < 0.05; **, p < 0.01; ***, p < 0.001 versus control group. [file JEV2-10-e12027-s005.pdf]

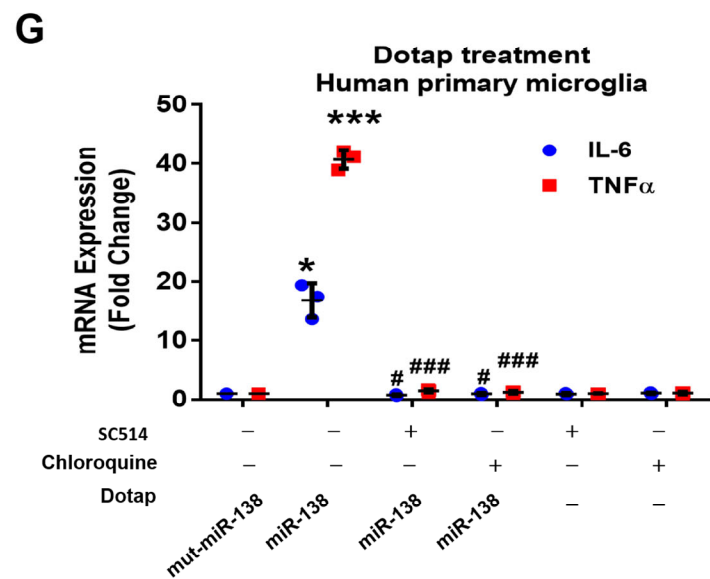

Supplement: Supplementary file 6 — SFigure 6. DOTAP miR‐138 activates microglia via the TLR7‐NF‐kB signaling pathway. (A) Real‐time PCR analysis of IL‐6 and TNF‐α in BV‐2 cells treated with EVs isolated from control or morphine stimulated astrocytes or ssRNA40. (B) Real time PCR analysis of IL‐6 and TNF‐α in BV‐2 cells transfected with DOTAP formulations of miR‐138 or mutant‐miR‐138. Real‐time PCR analysis of IL‐6 (C) and TNFα (D) in WT or TLR7 KO microglial cells transfected with Dotap‐miR138, DOTAP‐miR196 or LPS. IL‐6 (E) and TNFα (F) was assayed by ELISA in supernatants of WT or TLR7 KO microglial cells transfected with or without Dotap‐miR138, DOTAP‐miR196, or treated with LPS. (G) Real time PCR analysis of IL‐6 and TNF‐α in human primary microglial cells pretreated with endosomal TLR inhibiter Chloroquine, IKK‐2 inhibitor SC514 for 1 hr, followed by exposure to DOTAP‐miR‐138 and DOTAP‐mut‐miR‐138 for additional 4 hr. All data are presented as mean ± SD or SEM of three independent experiments. *, p < 0.05; **, p < 0.01; ***, p < 0.001 versus control group. [file JEV2-10-e12027-s006.pdf]
