# Supplementary material for: Morphine‐mediated release of miR‐138 in astrocyte‐derived extracellular vesicles promotes microglial activation
Source: J Extracell Vesicles. 2020 Nov 19;10(1):e12027. doi: 10.1002/jev2.12027 (PMC7710131; doi:10.1002/jev2.12027)
Supplement: Supplementary file 2 — SFigure 2. Characterization of EVs Isolated from Astrocyte Cultures. (A) Western blot characterization of mouse primary astrocyte EVs using the exosome marker antibodies specific for TSG101, CD63, and Alix. Calnexin was used as a control for cell debris contamination. (B) Size and particle distribution plots of isolated EVs from mouse primary astrocyte by ZetaView. The plot shows a peak size around 100 nm for the isolated EVs. (C) Western blot characterization of A172 astrocyte EVs with exosome marker antibodies specific for TSG101, CD63, and Alix. Calnexin was used as a control for cell debris contamination. (D) Size and particle distribution plots of isolated EVs from A172 astrocyte by ZetaView. The plot shows a peak size around 100 nm for the isolated EVs. (E) Western blot characterization of human primary astrocyte EVs with exosome marker antibodies specific for TSG101, CD63, and Alix. Calnexin was used as a control for cell debris contamination. (F) Size and particle distribution plots of isolated EVs from human primary astrocyte by ZetaView. The plot shows a peak size around 100 nm for the isolated EVs. (G) AFM image of EVs isolated from mouse primary astrocyte culture supernatants. All experiments were done for at least three independent times. [file JEV2-10-e12027-s002.pdf]

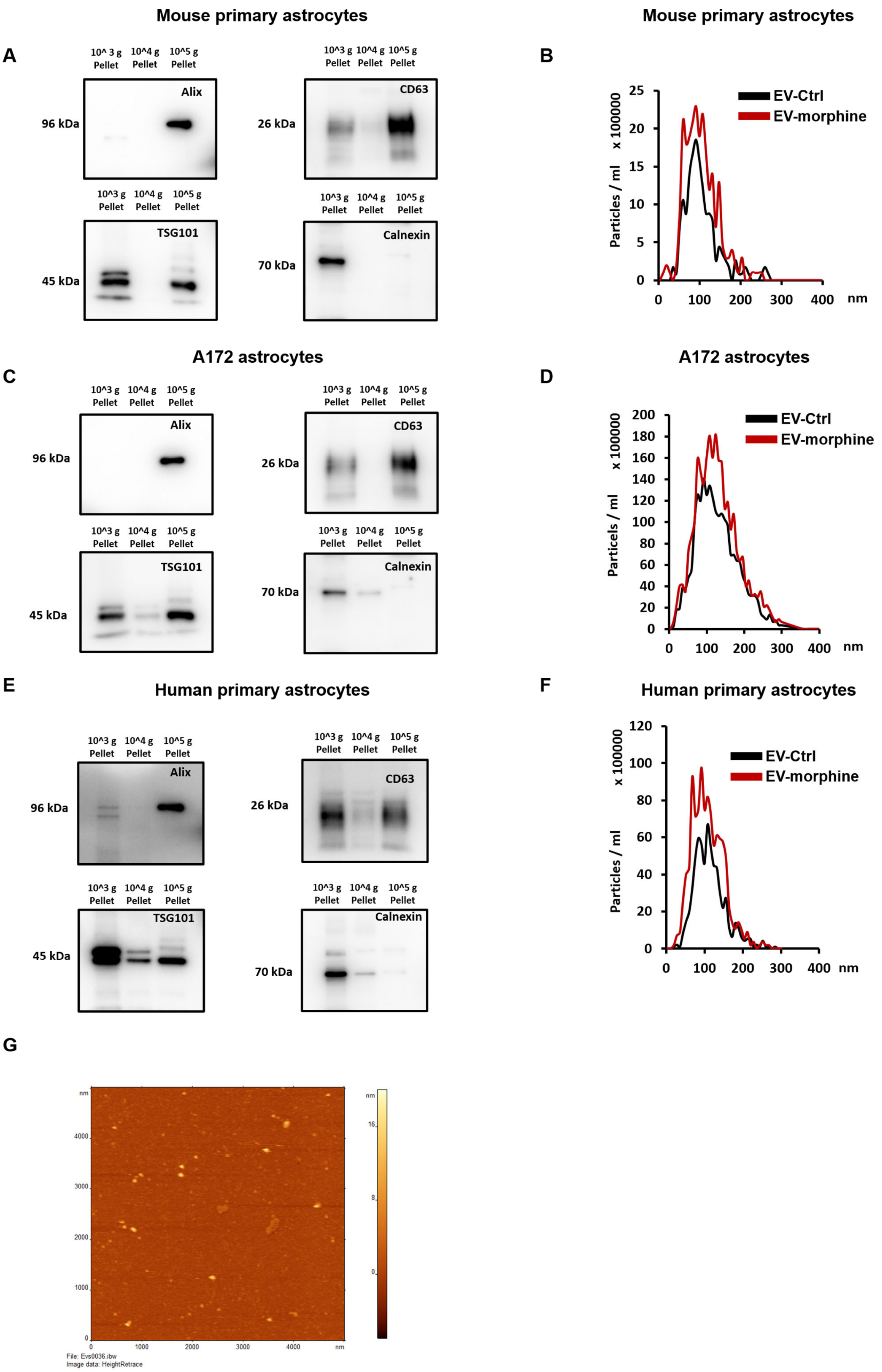
Mouse primary astrocytes
A
10^ 3 g Pellet
10^4 g Pellet
10^5 g Pellet
Alix
96 kDa
10^3 g Pellet
10^4 g Pellet
10^5 g Pellet
CD63
26 kDa
10^3 g Pellet
10^4 g Pellet
10^5 g Pellet
TSG101
45 kDa
10^3 g Pellet
10^4 g Pellet
10^5 g Pellet
Calnexin
70 kDa
Mouse primary astrocytes
B
EV-Ctrl
EV-morphine
Particles / ml x 100000
0
5
10
15
20
25
0
100
200
300
400
nm
A172 astrocytes
C
10^3 g Pellet
10^4 g Pellet
10^5 g Pellet
Alix
96 kDa
10^3 g Pellet
10^4 g Pellet
10^5 g Pellet
CD63
26 kDa
10^3 g Pellet
10^4 g Pellet
10^5 g Pellet
TSG101
45 kDa
10^3 g Pellet
10^4 g Pellet
10^5 g Pellet
Calnexin
70 kDa
A172 astrocytes
D
EV-Ctrl
EV-morphine
Particels / ml x 100000
0
20
40
60
80
100
120
140
160
180
200
0
100
200
300
400
nm
E
Human primary astrocytes
10^3 g Pellet
10^4 g Pellet
10^5 g Pellet
Alix
96 kDa
10^3 g Pellet
10^4 g Pellet
10^5 g Pellet
CD63
26 kDa
10^3 g Pellet
10^4 g Pellet
10^5 g Pellet
TSG101
45 kDa
10^3 g Pellet
10^4 g Pellet
10^5 g Pellet
Calnexin
70 kDa
F
Human primary astrocytes
EV-Ctrl
EV-morphine
Particles / ml x 100000
0
20
40
60
80
100
120
0
100
200
300
400
nm
G
nm
4000
3000
2000
1000
0
0
1000
2000
3000
4000
nm
nm
16
8
0
File: Evs0036.ibw
Image data: HeightRetrace
